# Piezo1 is the cardiac mechanosensor that initiates the cardiomyocyte hypertrophic response to pressure overload in adult mice

**DOI:** 10.1038/s44161-022-00082-0

**Published:** 2022-06-13

**Authors:** Ze-Yan Yu, Hutao Gong, Scott Kesteven, Yang Guo, Jianxin Wu, Jinyuan Vero Li, Delfine Cheng, Zijing Zhou, Siiri E. Iismaa, Xenia Kaidonis, Robert M. Graham, Charles D. Cox, Michael P. Feneley, Boris Martinac

**Affiliations:** 1 Molecular Cardiology and Biophysics Division, Victor Chang Cardiac Research Institute, Sydney, New South Wales Australia; 2 Cardiac Physiology and Transplantation Division, Victor Chang Cardiac Research Institute, Sydney, New South Wales Australia; 3 St Vincent’s Clinical School, Faculty of Medicine, University of New South Wales, Sydney, New South Wales Australia; 4 Department of Cardiology, St Vincent’s Hospital, Sydney, New South Wales Australia

**Keywords:** Cardiac hypertrophy, Ion channel signalling

## Abstract

Pressure overload-induced cardiac hypertrophy is a maladaptive response with poor outcomes and limited treatment options. The transient receptor potential melastatin 4 (TRPM4) ion channel is key to activation of a Ca^2+^/calmodulin-dependent kinase II (CaMKII)-reliant hypertrophic signaling pathway after pressure overload, but TRPM4 is neither stretch-activated nor Ca^2+^-permeable. Here we show that Piezo1, which is both stretch-activated and Ca^2+^-permeable, is the mechanosensor that transduces increased myocardial forces into the chemical signal that initiates hypertrophic signaling via a close physical interaction with TRPM4. Cardiomyocyte-specific deletion of Piezo1 in adult mice prevented activation of CaMKII and inhibited the hypertrophic response: residual hypertrophy was associated with calcineurin activation in the absence of its usual inhibition by activated CaMKII. Piezo1 deletion prevented upregulation of the sodium–calcium exchanger and changes in other Ca^2+^ handling proteins after pressure overload. These findings establish Piezo1 as the cardiomyocyte mechanosensor that instigates the maladaptive hypertrophic response to pressure overload, and as a potential therapeutic target.

## Abstract (web summary)

Yu et al. show that Piezo1 is the stretch-activated mechanosensor that provides the calcium source to activate TRPM4 and the downstream CaMKII-HDAC4-MEF2 pathway, a key mediator in the cardiomyocytes’ hypertrophic response in pressure overload models.

## Main

Despite advances in cardiovascular medicine over the last 30 yr, pathological left ventricular (LV) hypertrophy (LVH) secondary to pressure overload resulting from hypertension or aortic stenosis remains a powerful independent predictor of cardiovascular mortality and morbidity^1–4^. Thus far, the only treatment available for this condition is blood pressure reduction with anti-hypertensive medications or replacement of a stenotic aortic valve. These strategies do not fully reverse the pathological remodeling that occurs once LVH is established. Understanding the molecular mechanisms that drive LVH^5–8^ in response to pressure overload may open avenues to anti-hypertrophic therapies.

The two principal stimuli involved in LVH development are neuroendocrine hormones that activate Gq receptors (for example, angiotensin II) and mechanical forces (for example, pressure overload)^9^. These stimuli are thought to activate two distinct Ca^2+^-dependent signaling cascades that ultimately result in LVH: the calcineurin-nuclear factor of activated T cells (NFAT)-GATA4 pathway and the Ca^2+^/calmodulin-dependent protein kinase II (CaMKII)-histone deacetylase 4 (HDAC4)-myocyte enhancer factor 2 (MEF2) pathway. Our previous experimental work has demonstrated that the development of LVH in response to transverse aortic constriction (TAC), the most common surgical model of pressure overload, is dependent on activation of the CaMKII-HDAC4-MEF2 (refs. ^10–12^) pathway but does not require Gq receptor or calcineurin activation^6^.

Moreover, we have shown recently that the Ca^2+^-activated TRPM4 ion channel acts as a positive regulator of pressure overload-induced cardiac hypertrophy, playing a key role in the activation of the CaMKII-HDAC4-MEF2 pathway^7^. Given that TRPM4 is not activated by membrane stretch^13^ and that Gq receptor activation is not required, the question remains as to the identity of the molecule at the start of the hypertrophic signaling cascade that senses changes in mechanical load within the myocardium and transduces that mechanical signal into a chemical signal that activates TRPM4 and in turn the CaMKII-HDAC4-MEF2 pathway.

A prime candidate to act upstream of TRPM4 within this mechanosensory signaling cascade that drives LVH is the Ca^2+^-permeable mechanosensitive ion channel, Piezo1. Since the discovery and cloning of Piezo channels in 2010 (ref. ^14^), this family of structurally distinct ion channels has emerged as key sensors of biomechanical forces in the cardiovascular system^15^. Despite the significant evidence for a key role of Piezo1 channels in vascular physiology and pathophysiology^16–18^, little is known about the role of Piezo1 in cardiac biology. A recent study has identified that Piezo1 is expressed at low levels in adult murine cardiomyocytes and mediates a small stretch-activated Ca^2+^ current in native adult cardiomyocytes^19^. Genetic ablation of Piezo1 using a cardiomyocyte-specific Cre driver during early cardiac development resulted in a mild dilated cardiomyopathy that worsened as the mice aged^19^. However, whether Piezo1 expressed in cardiomyocytes is the mechanosensor responsible for initiating the CaMKII-HDAC4-MEF2 hypertrophic signaling cascade to induce LVH in response to pressure overload remains to be determined.

Here, using mice expressing a Piezo1 fusion protein (Piezo1–tdTomato) and a cardiomyocyte-specific inducible knockout (KO) (Piezo1 KO) mouse model, we investigate the role of Piezo1 in LVH induced by pressure overload. We demonstrate that in response to TAC-induced pressure overload, cardiomyocyte expression of Piezo1 increases, and that deletion of cardiomyocyte Piezo1 completely prevents activation of the CaMKII-HDAC4-MEF2 pathway and inhibits the LVH observed in response to TAC. Moreover, we show that Piezo1 not only colocalizes with TRPM4 in cardiomyocytes but that these channels physically interact, providing a basis for their functional coupling. Loss of Piezo1 prevents the altered expression of several critical Ca^2+^ handling proteins, including TRPM4 and the sodium–calcium exchanger (NCX), that are associated with pressure overload-induced LVH. Taken together, our findings demonstrate that Piezo1 is the mechanosensor that transduces the increased myocardial forces caused by pressure overload into a chemical signal that activates the hypertrophic signaling cascade resulting in pathological LVH.

## Results

### TAC induces LVH in wild-type (WT) and Piezo1^P1-tdT/P1-tdT^ mice

To test whether Piezo1 was involved in the signaling cascade that drives pathological LVH, we employed *Piezo1*^P1-tdT/P1-tdT^ mice that expressed a Piezo1–tdTomato fusion protein from the *Piezo1* locus^17^. This reporter mouse allowed us to use Piezo1 fusion proteins to probe Piezo1 expression using a specific mCherry antibody. We took this approach because there are no specific commercial mouse anti-Piezo1 antibodies. We performed TAC on *Piezo1*^P1-tdT/P1-tdT^ mice and their WT littermates (WTLs).

**Extended Data Fig. 1:**
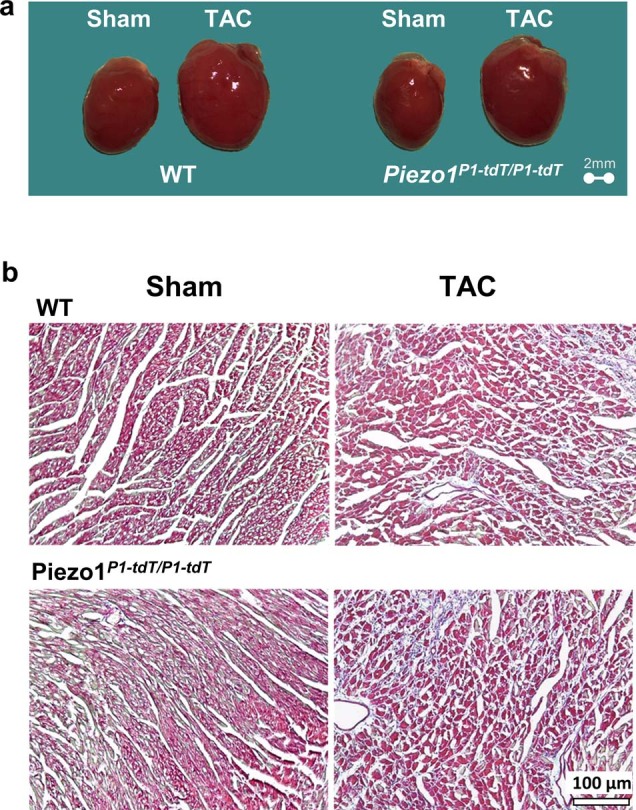
Cardiac enlargement and cardiac fibrosis were detected 14 days after TAC. (**a**) Representative photos of hearts from wild type littermates (WTLs) and *Piezo1*^*P1-tdT/P1-tdT*^ mice at 14 days after sham or TAC. (**b**) Representative photos of cardiac fibrosis, evaluated by Masson’s trichrome staining of LV tissue from WTLs mice subjected to TAC versus sham-operated controls. Scale bar = 100 µm.

TAC increased LV systolic pressure (LVSP) by ~57 mmHg in both WTLs and *Piezo1*^*P1-tdT/P1-tdT*^ mice, when compared with their respective sham-operated controls at 14 d (Extended Data Table 1; both *P* < 0.001). This significant pressure overload with TAC resulted in enlarged hearts (Extended Data Table 1 and Extended Data Fig. 1a) and significant LVH after 14 d in both groups, when compared with sham-operated controls. The degree of LVH did not differ between WTLs and *Piezo1*^*P1-tdT/P1-tdT*^ mice, whether LVH was assessed by echocardiographically determined LV mass, wall thickness (*h*) or wall thickness to chamber radius ratio (*h*/*r*), or by post-mortem LV weight (LVW), whether normalized to body weight (LVW/BW) or tibial length (LVW/TL) (Extended Data Table 1). Consistent with the development of pathological LVH, TAC was associated with increased cardiac fibrosis (*P* < 0.001; Extended Data Table 1 and Extended Data Fig. 1b) and enhanced collagen III (*Col3a1*) expression (*P* < 0.001; Extended Data Table 1) in both WTLs and *Piezo1*^P1-tdT/P1-tdT^ mice.

Notably, TAC-induced LVH at 14 d was not associated with any evidence of LV dysfunction in either group: there were no significant changes in heart rate, echocardiographically determined LV end-diastolic volume (LVEDV), LV end-systolic volume, LV ejection fraction, cardiac output, the derivative of pressure (*P*) over time (*t*) d*P*/d*t*_max_, d*P*/d*t*_min_, lung weight or lung weight to body weight ratio (Extended Data Table 1), indicating that our TAC model is a model of pressure overload LVH without ventricular decompensation or heart failure, as reported previously^6,7^.

### Markers of LVH induction in WT and Piezo1^P1-tdT/P1-tdT^ mice

Consistent with the early induction of hypertrophic signaling after TAC^6,7^, gene expression of atrial natriuretic peptide (ANP, *Nppa*), brain natriuretic peptide (BNP, *Nppb*) and α-skeletal actin (α-SA, *Acta1*) was increased significantly 48 h after TAC, preceding the development of significant LVH (Supplementary Table 1), in both whole LV tissue and isolated LV cardiomyocytes, with no significant differences between WTLs and *Piezo1*^*P1-tdT/P1-tdT*^ hearts (Extended Data Table 2). The increased expression in whole LV tissue and isolated LV cardiomyocytes persisted 14 d after TAC in WTLs and *Piezo1*^*P1-tdT/P1-tdT*^ mice (Extended Data Table 2).

### Pressure overload induces Piezo1 upregulation

**Fig. 1:**
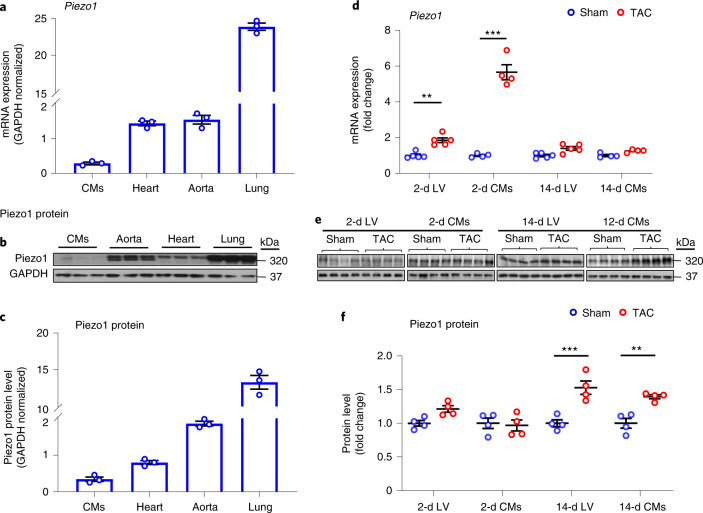
Piezo1 expression was upregulated in response to LV pressure overload in *Piezo1*^*P1-tdT/P1-tdT*^ mice. **a**–**c**, Relative Piezo1 mRNA expression (*n* = 3 per group) (**a**), representative western blots of Piezo1 protein expression (**b**) and quantitative densitometric analysis of 320-kDa Piezo1 protein expression from isolated cardiomyocytes (CMs), whole heart, aorta and lung tissues collected from *Piezo1*^*P1-tdT/P1-tdT*^ mice under baseline conditions (**c**). Relative Piezo1 mRNA and protein expression from isolated CMs, heart, aorta and lung were normalized to GAPDH (*n* = 3 per group). **d**, Relative Piezo1 mRNA expression in LV tissue and in LV CMs 2 d and 14 d after TAC or sham surgery (*n* = 5 per group) from *Piezo1*^*P1-tdT/P1-tdT*^ mice. **e**, Representative western blots of Piezo1 protein expression in LV tissue and in LV CMs. **f**, Quantitative densitometric analysis of 320-kDa Piezo1 protein expression from LV tissue and LV CMs 2 d and 14 d after TAC from *Piezo1*^*P1-tdT/P1-tdT*^ mice (*n* = 4 per group). Relative Piezo1 mRNA and protein expression in the LV tissue and CMs were normalized to GAPDH and calculated as fold change relative to sham in the 2-d and 14-d groups, respectively. Results are presented as mean ± s.e.m.; two-way ANOVA with Tukey’s post hoc test for multiple comparisons was used to assess effects of genotype, surgery and genotype by surgery interaction for **d**; and Welch’s *t*-test, two-tailed, was used for **f**; **P < 0.01, ***P < 0.001 versus sham-operated groups for respective genotype. Source data

Consistent with previous reports^14,16^, baseline *Piezo1* messenger RNA levels were low in isolated LV cardiomyocytes (Fig. 1a). We used the Piezo1–tdTomato fusion protein to measure Piezo1 protein levels. The Piezo1–tdTomato fusion protein (~320 kDa) was detected in both whole heart tissue and isolated cardiomyocytes (Fig. 1b). Under baseline conditions, Piezo1 protein levels were low in isolated cardiomyocytes, particularly when compared with the aorta and lung (Fig. 1c).

At 2 d after TAC, however, *Piezo1* mRNA expression increased, ~2-fold in LV tissue (*P* < 0.01) and ~6-fold in isolated LV cardiomyocytes (*P* < 0.001; Fig. 1d), when compared with sham-operated hearts. The upregulation of *Piezo1* mRNA expression was not maintained 14 d after TAC, by which time mRNA expression was similar to sham-operated controls (Fig. 1d). In contrast, Piezo1 protein levels did not increase significantly in either LV tissue or isolated cardiomyocytes 2 d after TAC (Fig. 1e,f) but did increase significantly 14 d after TAC in both LV tissue (~1.6-fold; *P* < 0.001) and isolated cardiomyocytes (~1.4-fold*; P* < 0.01) (Fig. 1e,f).

### Targeted deletion of Piezo1 from adult cardiomyocytes

**Extended Data Fig. 2:**
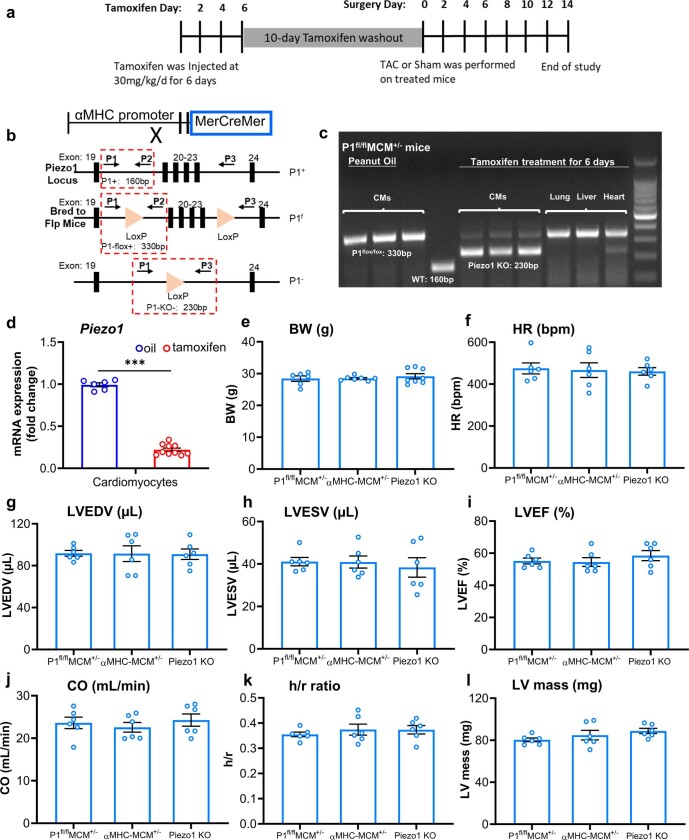
Cardiomyocyte-specific tamoxifen inducible Piezo1 KO. (**a**) Experimental design timeline for tamoxifen induction, TAC surgery and serial echocardiograms. (**b**) A schematic diagram showing the generation of cardiomyocyte-specific *Piezo1* knockout (KO) mice. (**c**) Confirmation of Cre-recombination by PCR of cardiac genomic DNA extracted from isolated CMs from P1^fl/fl^MCM^+/-^ hearts treated with tamoxifen or with peanut oil (n = 3/group). (**d**) Piezo1 mRNA expression confirmed by qPCR measured from isolated CMs from P1^fl/fl^MCM^+/-^ hearts treated with tamoxifen (Piezo1 KO, n = 10/group) or with peanut oil (n = 6) using GAPDH as an internal control. (**e**-**l**) Echocardiographic measurements following tamoxifen treatment of αMHC-MCM^+/-^, Piezo1 KO mice and P1^fl/fl^MCM^+/-^ controls showing (**e**) BW, Body weight. (**f**) HR, heart rate. (**g**) LVEDV, LV end-diastolic volume. (**h**) LVESV, LV end-systolic volume. (**i**) LVEF, LV ejection fraction. (**j**) CO, cardiac output. (**k**) h/r, LV wall thickness to chamber radius ratio. (**l**) LV mass. Results are presented as mean ± SEM, Welch’s T-test, two-tailed was used for d and One-way ANOVA with Tukey’s post-hoc test for multiple comparisons for **e-l**, ***p < 0.001 vs. peanut oil treated cardiomyocytes. Source data

**Extended Data Fig. 3:**
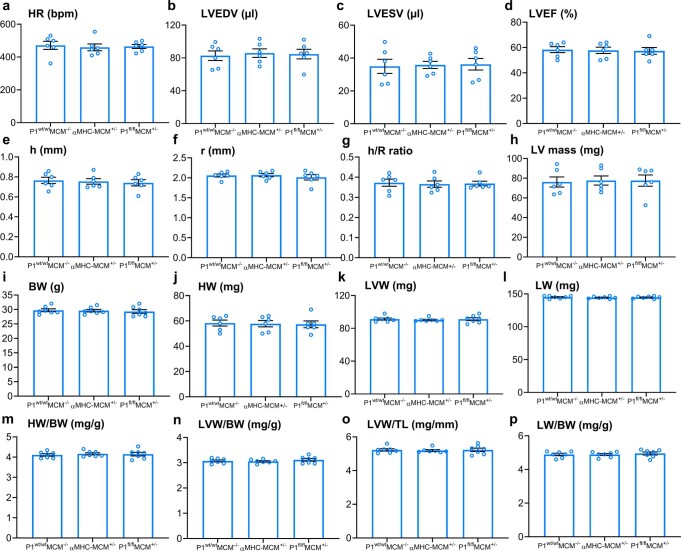
The Piezo1^fl/fl^MCM^+/-^ mice show normal cardiac function and morphology under baseline conditions. (**a-h**) Echocardiographic analysis of (**a**) HR, heart rate, (**b**) LVEDV, LV end-diastolic volumes, (**c**) LVESV, LV end-systolic volumes, (**d**) LVEF, LV ejection fraction, (**e**) h, LV wall thickness, (**f**) r, chamber radius, (**g**) h/r ratio, LV wall thickness to chamber radius ratio and (**h**) LV mass, n = 6/group. (**i-p**) Anatomic analysis of (**l**) BW, body weight, (**j**) HW, heart weight, (**k**) LVW, left ventricular weight, (**l**) LW, lung weight, (**m**) HW/BW, heart weight to body weight ratio, (**n**) LVW/BW, left ventricular weight to body weight ratio, (**o**) LVW/TL, left ventricular weight to tibia length ratio, (**p**) LW/BW, lung weight to body weight ratio, n = 7/group. Results are presented as mean ± SEM, one-way ANOVA with Tukey’s post-hoc test for multiple comparisons were used for **a-p**. Source data

To further investigate whether Piezo1 plays an important role in the induction of LVH secondary to pressure overload, we generated an inducible cardiomyocyte-specific Piezo1 KO mouse (Piezo1 KO) that permitted deletion of Piezo1 from adult cardiomyocytes (8+ weeks old) (Extended Data Fig. 2a,b). Tamoxifen-inducible α-MHC-MerCreMer transgenic mice (Cre transgene under control of the α-myosin heavy-chain (αMHC) (*Myh6*) promoter^20^) were crossed with Piezo1^fl/fl^ mice, and offspring backcrossed until P1^fl/fl^MCM^+/−^ mice were generated (Methods and Extended Data Fig. 2b,c). To account for the potential nonspecific Cre-recombinase-mediated cardiotoxicity^21^, P1^wt/wt^MCM^+/−^ mice were designated as controls for Cre. P1^fl/fl^MCM^+/−^ male mice aged 8–10 weeks were indistinguishable in cardiac function and anatomical parameters from age- and sex-matched P1^wt/wt^MCM^+/−^ and P1^wt/wt^MCM^−/−^ control mice (Extended Data Fig. 3).

**Extended Data Fig. 4:**
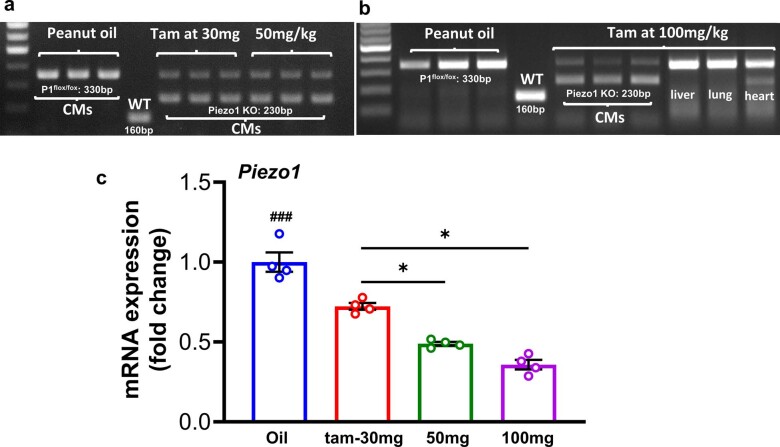
Tamoxifen induced recombinase activity in a dose-dependent manner. Confirmation of Cre-recombination by PCR of cardiac genomic DNA extracted from isolated CMs from P1^fl/fl^MCM^+/-^ hearts 10 days after the last injection with tamoxifen (tam) at 30 mg, 50 mg and 100 mg/kg/d for 3 consecutive days or with peanut oil (n = 3/group). (**c**) Piezo1 mRNA expression confirmed by qPCR in isolated CMs from P1^fl/fl^MCM^+/-^ hearts from tamoxifen or peanut oil treated mice (n = 4/group) using GAPDH as an internal control. Results are presented as mean ± SEM, one-way ANOVA with Tukey’s post-hoc test for multiple comparisons were used for c, ### *p* < 0.001 vs. all tamoxifen-treated groups, *p < 0.01, ***p < 0.001 vs. tamoxifen 30 mg/kg/d group. Source data

To establish a dosing regimen of tamoxifen that maximized Cre-recombinase activity at the Piezo1 locus but minimized tamoxifen-induced cardiotoxicity^21,22^, we injected different concentrations of tamoxifen into 8-week-old male P1^fl/fl^MCM^+/−^ and P1^wt/wt^MCM^+/−^ mice on 3 consecutive days, and allowed 10 d for them to recover. We injected the same volume of peanut oil into P1^fl/fl^MCM^+/−^ mice as the treatment control. We observed tamoxifen-induced Cre-recombinase activity in a dose-dependent manner (Extended Data Fig. 4a–c). Piezo1 deletion was observed in cardiomyocytes of mice injected with tamoxifen but not peanut oil. P1^fl/fl^MCM^+/−^ mice treated with tamoxifen at 30 mg kg^−1^ d^−1^ exhibited normal heart structure and function when compared with mice treated with peanut oil (Supplementary Table 2). Higher doses of tamoxifen, 50 mg kg^−1^ d^−1^ or 100 mg kg^−1^ d^−1^, caused LV dilatation with impaired contraction (Supplementary Table 2). Consequently, a tamoxifen dose of 30 mg kg^−1^ d^−1^ was used in subsequent experiments.

Tamoxifen (30 mg kg^−1^ d^−1^ for 6 consecutive days) treatment of P1^fl/fl^MCM^+/−^ mice produced efficient inducible deletion of *Piezo1* when measured 10 d after the last injection (Extended Data Fig. 2). We confirmed successful Cre-recombination using PCR amplification of cardiac genomic DNA^23^. As shown in Extended Data Fig. 2c, Cre-mediated recombination of *Piezo1* was detected only in the heart, with no excision identified in liver or lung tissues. A stronger signal was identified in isolated cardiomyocytes when compared with whole heart tissue from tamoxifen-treated P1^fl/fl^MCM^+/−^ mice, and no excision was evident in cardiomyocytes isolated from control mice treated with peanut oil (Extended Data Fig. 2c). Congruent with these results, and consistent with previous findings of the relative efficiency for the αMHC-MerCreMer construct^20,24^, expression of Piezo1 mRNA was reduced by approximately 78% in isolated cardiomyocytes from tamoxifen-treated P1^fl/fl^MCM^+/−^ mice (Piezo1 KO mice, *P* < 0.001) when compared with mice injected with peanut oil (Extended Data Fig. 2c,d). These findings confirm that tamoxifen at 30 mg kg^−1^ d^−1^ for 6 d induced specific deletion of Piezo1 from cardiomyocytes.

**Fig. 2:**
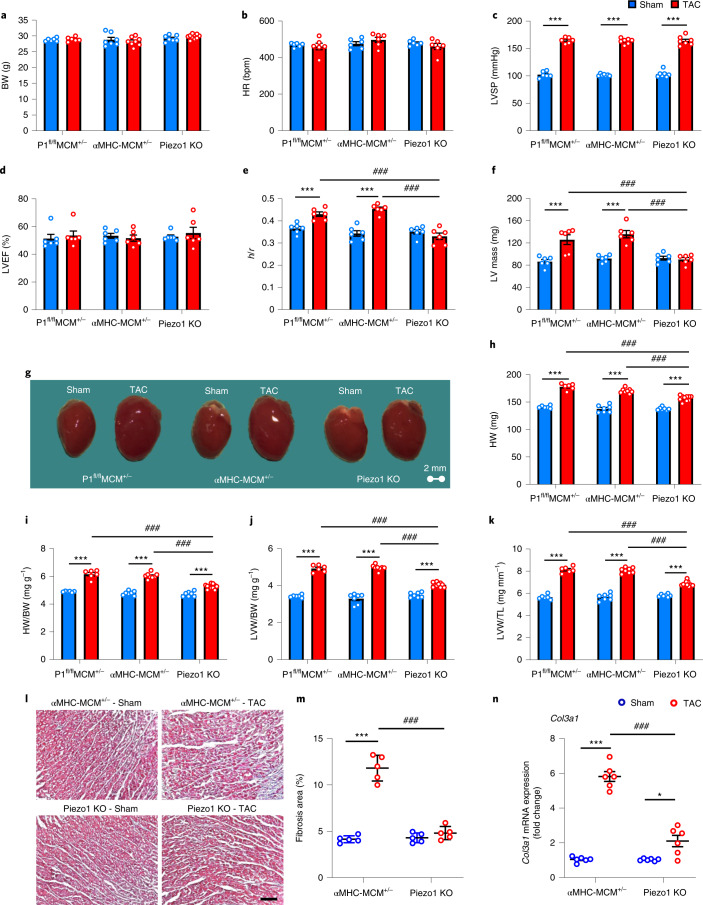
Piezo1 KO mice exhibit less LVH in response to pressure overload. **a**, Body weight (BW). **b**, Heart rate (HR). **c**, LVSP. **d**, LV ejection fraction (LVEF). **e**, LV wall thickness to chamber radius ratio (*h*/*r*). **f**, LV mass. **g**, Representative photos indicate heart size differences 14 d after TAC or sham surgery in αMHC-MCM^+/−^ mice, Piezo1 KO mice and P1^fl/fl^MCM^+/−^ controls. **h**–**k**, Anatomical analyses (*n* = 6–10 per group as indicated by individual data points) of heart weight (HW) (**h**), heart weight to body weight ratio (HW/BW) (**i**), LV weight to body weight ratio (LVW/BW) (**j**) and LV weight to tibia length (LVW/TL) (**k**). **l**, Representative micrographs of Masson’s trichrome staining of LV tissue; scale bar, 200 µm. **m**, Quantitation of Masson’s trichrome staining of LV tissue 14 d after TAC or sham surgery (*n* = 5 per group). **n**, Relative Collagen III (*Col3a1*) mRNA expression 14 d after TAC or sham surgery (*n* = 6 per group). The mRNA relative expression was normalized with GAPDH and calculated as fold change relative to sham in P1^fl/fl^MCM^+/−^ mice treated with peanut oil. Results are presented as mean ± s.e.m.; two-way ANOVA with Tukey’s post hoc test for multiple comparisons was used to assess effects of genotype, surgery and genotype by surgery interaction for **a**–**f** and **h**–**n**; *P < 0.05, ***P < 0.001 versus sham-operated groups for respective genotype; ^###^*P* < 0.001, versus TAC groups. Source data

**Extended Data Fig. 5:**
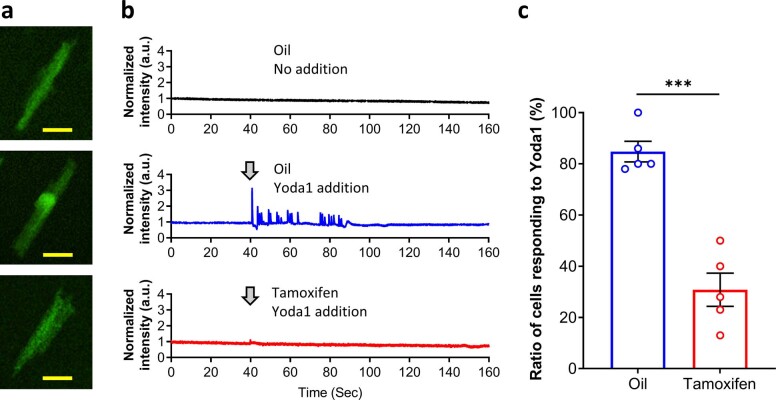
Piezo1-mediated Ca^2+^ response in isolated cardiomyocytes from P1^fl/fl^MCM^+/-^ and Piezo1 KO left ventricle. (**a**) Exemplary Ca^2+^ images of single mouse LV cardiomyocyte. Scale bar = 50 µm. (**b**) Representative normalized intensity traces of the cardiomyocytes during experiment. Gray arrows indicating Yoda1 addition (final concentration 30 μM). Top: peanut oil-treated P1^fl/fl^MCM^+/-^ control mice without Yoda1 addition; middle: peanut oil-treated P1^fl/fl^MCM^+/-^ mice with Yoda1 addition; bottom: tamoxifen-treated P1^fl/fl^MCM^+/-^ mice with Yoda1 addition, in both (**a**) and (**b**). (**c**) The ratio of cardiomyocytes responding to Yoda1, compared between peanut oil-treated and tamoxifen-treated P1^fl/fl^MCM^+/-^ mice. n = 5 wells in a 96-well plate in each group. In total 50 cells from 2 hearts in peanut oil-treated group; 72 cells from 2 hearts in tamoxifen-treated group. Results are presented as mean ± SEM with scatter plot, unpaired two-tailed student’s T-test, ***p < 0.001 vs. peanut oil-treated cardiomyocytes. Source data

To confirm the deletion of Piezo1 in mouse cardiomyocytes, we used isolated LV cardiomyocytes from both peanut oil-treated and tamoxifen-treated P1^fl/fl^MCM^+/−^ mice to investigate the cell response to the Piezo1-specific agonist Yoda1 using Ca^2+^ imaging. Only quiescent cardiomyocytes without basal Ca^2+^ oscillations were analyzed because spontaneous Ca^2+^ waves are known to be an indication of Ca^2+^ overload^25^. Extended Data Fig. 5a shows exemplar images of Cal520-loaded single LV cardiomyocytes from peanut oil-treated and tamoxifen-treated mice. Extended Data Fig. 5b shows representative normalized intensity traces of the cardiomyocytes from peanut oil-treated P1^fl/fl^MCM^+/−^ mice without (black trace) or with Yoda1 addition (blue trace), as well as tamoxifen-treated mice with Yoda1 addition (red trace). Yoda1 (final concentration 30 μM (ref. ^19^)) was added to the cells at 40 s. Yoda1 elicited Ca^2+^ transients in significantly more cardiomyocytes from peanut oil-treated P1^fl/fl^MCM^+/−^ mice (84.8 %) compared with the cardiomyocytes from tamoxifen-treated P1^fl/fl^MCM^+/−^ mice (30.8 %) (*P* < 0.001; Extended Data Fig. 5c). This decreased excitability of mouse LV cardiomyocytes in response to Yoda1 treatment reflects the loss of Piezo1 expression and perhaps a degree of mosaicism in the deletion.

Next, we assessed whether tamoxifen-induced deletion of Piezo1 in cardiomyocytes had any impact on baseline cardiac function and structure. To do this, we treated P1^wt/wt^MCM^+/−^ mice with the same tamoxifen dose (30 mg kg^−1^ d^−1^) as a control for Cre expression (termed α-MHC-MCM^+/−^ mice). We performed echocardiographic measurements 10 d after mice received their last injection of tamoxifen for Piezo1 KO mice, α-MHC-MCM^+/−^ mice and P1^fl/fl^MCM^+/−^ control mice (peanut oil-injected). The results from this analysis showed that there were no significant differences in body weight (Extended Data Fig. 2e), heart rate (Extended Data Fig. 2f), cardiac function (Extended Data Fig. 2g–j) or LV morphology between any of the three groups (Extended Data Fig. 2k,l).

### Deletion of cardiomyocyte Piezo1 inhibits hypertrophy

TAC or sham surgery was performed on Piezo1 KO mice, α-MHC-MCM^+/−^ mice and P1^fl/fl^MCM^+/−^ control mice. After 14 d, body weight, heart rate, LV end-diastolic and end-systolic volumes, ejection fraction and d*P*/d*t* were not significantly different between TAC or sham-operated animals, or between the three genotypes (Fig. 2 and Extended Data Table 3). Importantly, TAC induced the same increase in LVSP in all three genotypes (Fig. 2c). Echocardiographic indices of LVH—LV wall thickness to chamber radius ratio (*h*/*r*) and LV mass—increased significantly (both *P* < 0.001) after TAC in both α-MHC-MCM^+/−^ mice and P1^fl/fl^MCM^+/−^ control mice (Fig. 2e,f), but these changes were absent in Piezo1 KO mice after TAC (Fig. 2e,f). Similarly, post-mortem indices of LVH—heart weight and LV weight, whether normalized to body weight or tibial length—increased very significantly (all *P* < 0.001) 14 d after TAC in both α-MHC-MCM^+/−^ mice and P1^fl/fl^MCM^+/−^ control mice, but Piezo1 KO mice exhibited significant inhibition of the hypertrophic response to TAC (Fig. 2g–k): for example, the average increase in the LV weight/tibial length ratio after TAC was 62% lower in Piezo1 KO mice (*P* < 0.001).

### Deletion of cardiomyocyte Piezo1 inhibits fibrotic changes

Pathological LVH is associated with cardiac fibrosis including upregulated collagen expression and deposition. We evaluated interstitial cardiac fibrosis in response to pressure overload 14 d after TAC in Piezo1 KO hearts and α-MHC-MCM^+/−^ hearts using Masson’s trichrome staining (Fig. 2l). There was increased interstitial cardiac fibrosis in α-MHC-MCM^+/−^ TAC hearts when compared with their sham controls (*P* < 0.001), but there was no increase in cardiac fibrosis in Piezo1 KO hearts after TAC (Fig. 2l,m). Consistent with these findings, Collagen III (*Col3a1*) mRNA expression increased ~6-fold in α-MHC-MCM^+/−^ TAC hearts when compared with their sham controls (*P* < 0.001), but a more attenuated response was observed in Piezo1 KO hearts after TAC when compared with sham-operated hearts (~2-fold; *P* < 0.05; Fig. 2n) and with αMHC-MCM^+/−^ TAC-operated hearts (*P* < 0.001; Fig. 2n).

### The hypertrophic gene program requires Piezo1

**Fig. 3:**
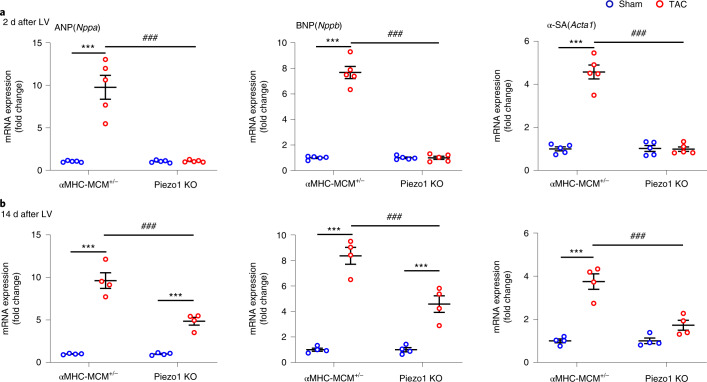
Comparison of gene expression of LVH markers in response to TAC-induced pressure overload in Piezo1 KO and control αMHC-MCM^+/−^ mice. **a**, Relative mRNA expression of ANP (*Nppa*), BNP (*Nppb*) and α-SA (*Acta1*) in LV tissue 2 d after TAC compared with sham-operated mice (*n* = 5 per group). **b**, Relative mRNA expression of ANP (*Nppa*), BNP (*Nppb*) and α-SA (*Acta1*) in LV tissue 14 d after TAC compared with sham-operated mice (*n* = 4 per group). The mRNA relative expression was normalized with GAPDH and calculated as fold change relative to αMHC-MCM^+/−^ sham-operated hearts in 2-d and 14-d groups, respectively. Results are presented as mean ± s.e.m.; two-way ANOVA with Tukey’s post hoc test for multiple comparisons was used to assess effects of genotype, surgery and genotype by surgery interaction for **a** and **b**; ***P < 0.001 versus sham-operated groups for respective genotype; ^###^*P* < 0.001 versus TAC groups. Source data

**Extended Data Fig. 6:**
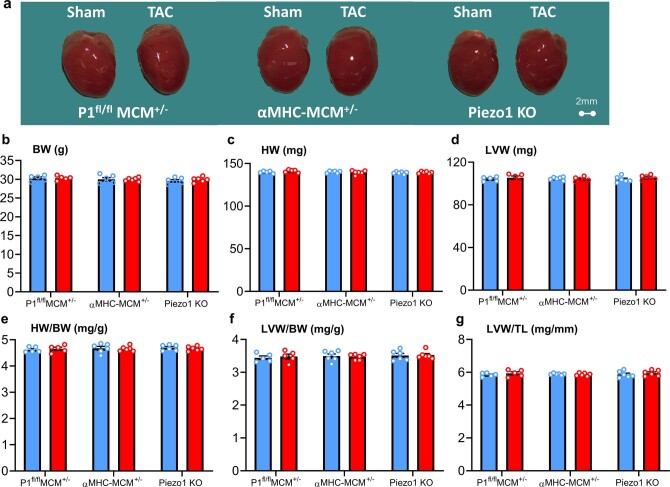
Piezo1 KO mice and control mice do not develop LVH in response to pressure overload 2 days after TAC. (**a**) Representative photos indicate normal heart size 2 days after TAC or sham surgery in αMHC-MCM^+/-^ mice, Piezo1 KO mice and P1^fl/fl^MCM^+/-^ controls. (**b-g**) Morphological analysis (n = 5–6/group) of (**b**) BW, body weight. (**c**) HW, heart weight. (**d**) LVW, left ventricular weight. (**e**) HW/BW, heart weight to body ratio. (**f**) LVW/BW, left ventricular weight to body weight ratio. (**g**) LVW/TL, left ventricular weight to tibia length ratio. Results are presented as mean ± SEM, one-way ANOVA with Tukey’s post-hoc test for multiple comparisons were used for **b-g**. Source data

Although LVH had not yet developed 2 d after TAC (Extended Data Fig. 6), induction of hypertrophic signaling in α-MHC-MCM^+/−^ hearts was already evident at this time, as was apparent from 5–10-fold increases in gene expression of ANP (*Nppa*), BNP (*Nppb*) and α-SA (*Acta1*) (all *P* < 0.001). However, this early response of markers of hypertrophic signaling was completely absent in Piezo1 KO hearts 2 d after TAC (Fig. 3a). The increased expression of these hypertrophy-associated genes was maintained 14 d after TAC in α-MHC-MCM^+/−^ hearts (~4–10-fold; all *P* < 0.001), while an attenuated late response was observed in Piezo 1 KO mice 14 d after TAC (~2–5-fold; *P* < 0.001 for both *Nppa* and *Nppb*, not significant for *Acta1*; Fig. 3b).

### Piezo1 activates CaMKII-HDAC4-MEF2 signaling

The cytoplasmic and the nuclear fractions of LV tissue were separated (Methods). Purity of the isolated fractions was confirmed by western blot analysis using antibodies against proteins specific for cytoplasmic (glyceraldehyde 3-phosphate dehydrogenase (GAPDH)) and nuclear (Histone H2B) fractions (Supplementary Fig. 1).

To examine whether Piezo1 influenced the baseline function of CaMKII, we performed western blots to quantify the amount of cytoplasmic or nuclear CaMKII in sham-operated α-MHC-MCM^+/−^ and Piezo1 KO hearts (Supplementary Fig. 2a). We found no difference in the protein level of CaMKII in either the cytoplasmic (Supplementary Fig. 2b) or nuclear fraction (Supplementary Fig. 2c) between the two genotypes of sham-operated hearts, suggesting that the deletion of Piezo1 in cardiomyocytes has no impact on the basal CaMKII protein expression in the absence of pressure overload.

**Fig. 4:**
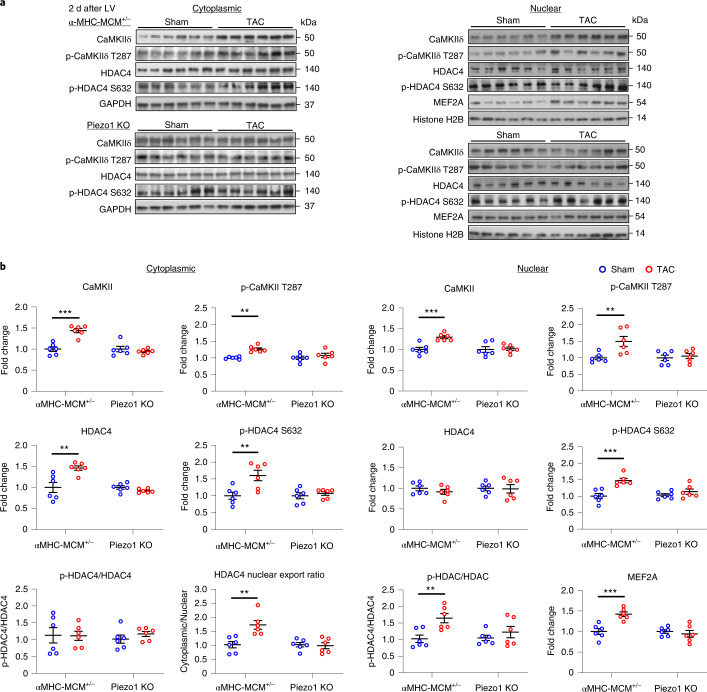
TAC-induced changes in the CaMKII-HDAC4-MEF2 signaling pathway in Piezo1 KO and αMHC-MCM^+/−^ mouse hearts. **a**, Representative western blots showing the expression of key proteins in the CaMKII-HDAC4-MEF2 signaling pathway in the cytoplasm (left panel) and nucleus (right panel). **b**, Cytoplasmic (left panel) and nuclear (right panel) quantitative data were normalized with GAPDH and Histone H2B, respectively. Fold changes and cytoplasmic/nuclear ratios were calculated relative to sham groups for each genotype (*n* = 6 per group). Results are presented as mean ± s.e.m.; Welch’s *t*-test, two-tailed, was used for comparisons between the two groups for **b**; **P < 0.01, ***P < 0.001 versus sham-operated group. Source data

As expected, α-MHC-MCM^+/−^ mice exhibited strong activation of the CaMKII-HDAC4-MEF2 hypertrophic signaling pathway 2 d after TAC (Fig. 4)^6,7^. The hallmarks of this activation are increased levels of both total and activated CaMKII in the cytoplasmic or nuclear fraction (*P* < 0.001, *P* < 0.01), which is auto-phosphorylated at threonine 287 (p-CaMKII T287)^26,27^; increased HDAC4 and phosphorylated-HDAC4 at the CaMKII-specific site; serine 632 (p-HDAC4 S632)^11^ in the cytoplasmic fraction (both *P* < 0.01); with no change in the ratio of cytoplasmic p-HDAC4/HDAC4. Analysis of α-MHC-MCM^+/−^ mice also showed that HDAC4 protein level was not changed in the nuclear fraction, but increased p-HDAC4 was detected (*P* < 0.001), leading to increase in the ratio of p-HDAC4 to HDAC4 (*P* < 0.01), and a resultant increase in the cytoplasmic to nuclear ratio of HDAC4 (*P* < 0.01), indicating nuclear export of HDAC4, with consequent de-repression of MEF2A (*P* < 0.001; Fig. 4).

Remarkably, Piezo1 KO mice failed to exhibit any evidence of activation of the CaMKII-HDAC4-MEF2 hypertrophic signaling pathway 2 d after TAC: the findings in Piezo1 KO mice 2 d after TAC were indistinguishable from those in their sham-operated controls (Fig. 4). These data indicate that Piezo1 is essential for activation of the CaMKII-HDAC4-MEF2 hypertrophic signaling pathway in response to pressure overload induced by TAC.

### Deletion of Piezo1 boosts calcineurin-NFAT signaling

**Fig. 5:**
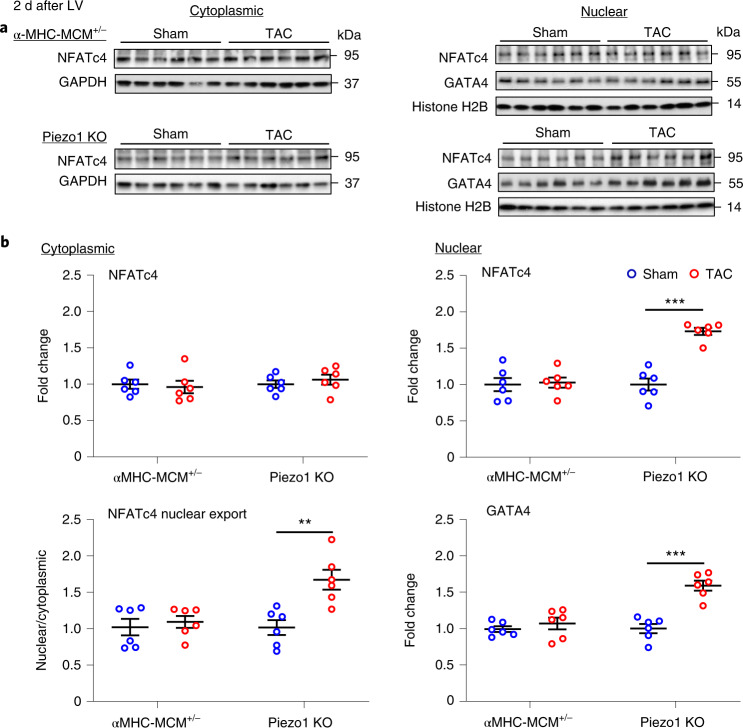
Calcineurin-NFAT signaling pathway in αMHC-MCM^+/−^ and Piezo1 KO mouse hearts 2 d post-TAC. **a**, Representative western blots showing the expression of NFAT and GATA4 in cytoplasm (left) and nucleus (right). **b**, Cytoplasmic and nuclear (right) quantitative data were normalized by GAPDH and Histone H2B, respectively (*n* = 6 per group). Fold changes and nuclear/cytoplasmic ratio were calculated relative to sham groups, in each genotype. Results are presented as mean ± s.e.m.; Welch’s *t*-test, two-tailed, was used for comparisons between the two groups for **b**; **P < 0.01, ***P < 0.001 versus sham-operated group. Source data

As expected, α-MHC-MCM^+/−^ mice exhibited no evidence of activation of the calcineurin-NFAT hypertrophic signaling pathway 2 d after TAC (Fig. 5)^6,7^. One explanation for this finding is that pressure overload activates CaMKII and activated CaMKII inhibits calcineurin activation^8^. Because Piezo1 KO mice exhibited no evidence of CaMKII activation in response to pressure overload, yet they exhibited incomplete inhibition of LVH, we postulated that the residual LVH was driven by calcineurin activation in the absence of its inhibition by CaMKII. The results obtained in Piezo1 KO mice 2 d after TAC supported this hypothesis: the hallmark of calcineurin activation, an increase in the nuclear to cytoplasmic NFAT ratio, indicating translocation of NFAT to the nucleus due to dephosphorylation by activated calcineurin, was clearly evident in Piezo1 KO mice 2 d after TAC (*P* < 0.01; Fig. 5a,b) but absent in both sham-operated controls and α-MHC-MCM^+/−^ mice subjected to TAC.

### Piezo1 is upstream of changes in Ca^2+^ handling proteins

We have demonstrated recently that the Ca^2+^-activated ion channel TRPM4 plays an important role in the activation of the Ca^2+^/calmodulin-dependent kinase, CaMKII, and thus the CaMKII-HDAC4-MEF2 hypertrophic signaling pathway^7^, but TRPM4 is neither stretch-activated nor Ca^2+^-permeable, whereas Piezo1 is both stretch-activated and Ca^2+^-permeable. To comprehensively investigate whether Piezo1 can modify the expression of proteins important in cardiomyocyte Ca^2+^ handling, we probed the expression of TRPM4; sarcoplasmic reticulum Ca^2+^ ATP-ase (SERCA2a); phospholamban (PLN); phosphorylated-PLN at the CaMKII-specific site, threonine 17 (p-PLN T17); NCX1; the L-type Ca^2+^ channel (Ca_v1.2_); and the T-type Ca^2+^ channel (Ca_v3.2_) in response to LV pressure overload.

**Fig. 6:**
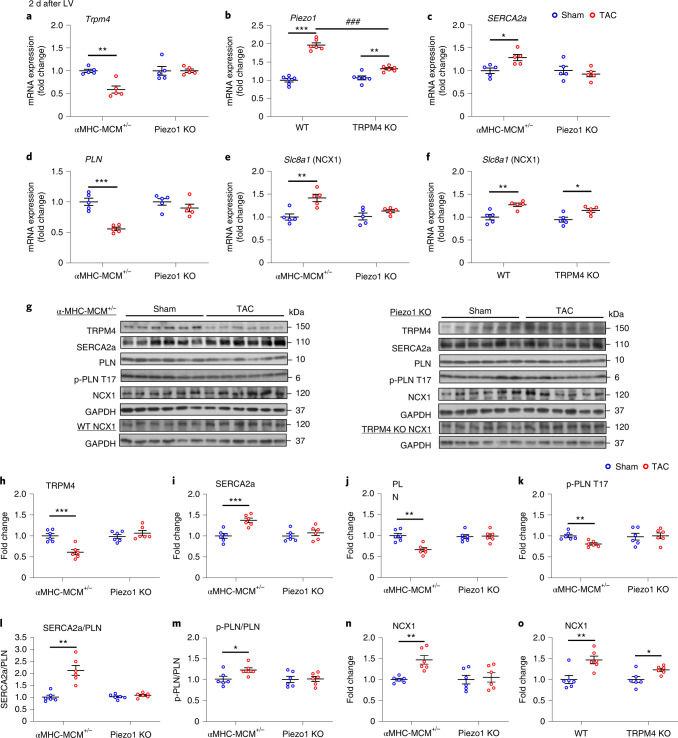
Gene expression and protein levels of selected Ca^2+^ handling molecules 2 d after TAC in Piezo1 KO and αMHC-MCM^+/−^ mouse hearts. **a**–**f**, Relative mRNA expression of: *Trpm4* (**a**), *Piezo1* (**b**), *SERCA2a* (**c**), *PLN* (**d**) and *Slc8a1* in Piezo1 KO (**e**) and *Slc8a1* in *Trpm4* KO (**f**) in LV tissue 2 d after sham or TAC. **g**, Representative western blots of TRPM4, SERCA2a, PLN, p-PLN T17 and NCX1 in LV tissue. **h**–**o**, Western blots from LV tissue after 2 d of TAC were used to quantify the protein levels of: TRPM4 (**h**), SERCA2a (**i**), PLN (**j**), p-PLN T17 (**k**), the ratio of SERCA2a/PLN (**l**), the ratio of p-PLN T17/PLN (**m**) and NCX1 in Piezo1 KO (**n**) and NCX1 in TRPM4 KO (**o**). Relative mRNA (*n* = 5 per group) and protein expression (*n* = 6 per group) in the LV tissue were normalized to GAPDH and calculated as fold change relative to the 2-d post-sham group, respectively. Results are presented as mean ± s.e.m.; two-way ANOVA with Tukey’s post hoc test for multiple comparisons was used to assess effects of genotype, surgery and genotype by surgery interaction for **a**–**f**; and Welch’s *t*-test, two-tailed, was used for **h**–**o**; *P < 0.05, **P < 0.01, ***P < 0.001 versus sham-operated groups for the respective genotypes; ^###^*P* < 0.001 versus TAC groups. Source data

α-MHC-MCM^+/−^ hearts showed significantly reduced expression of *Trpm*4 mRNA and TRPM4 protein 2 d after TAC (Fig. 6a,g,h; *P* < 0.01, *P* < 0.001), replicating our previous findings in WT mice^7^, but Piezo1 KO mice exhibited no change in *Trpm4* mRNA 2 d after TAC (Fig. 6a,g,h), indicating that Piezo1 is upstream of TRPM4, and mediates the response of TRPM4 to pressure overload.

Conversely, the upregulation of Piezo1 mRNA expression 2 d after TAC in WT hearts (*P* < 0.001; Fig. 6b) was not abolished in TRPM4 KO hearts (*P* < 0.01) 2 d after TAC, confirming that TRPM4 is downstream of Piezo1 in the response to pressure overload. Nevertheless, the magnitude of upregulation of Piezo1 mRNA 2 d after TAC was diminished significantly in TRPM4 KO mice (*P* < 0.001; Fig. 6b), suggesting that TRPM4 plays a key role in the feedback regulation of Piezo1 in response to pressure overload.

Cardiomyocyte Ca^2+^ handling requires the intimate interaction of numerous proteins, including SERCA2a, which mediates Ca^2+^ uptake into the sarcoplasmic reticulum, and NCX1, which enables Ca^2+^ extrusion into the extracellular space^28–31^. We next measured the mRNA and protein expression of SERCA2a and PLN, and its phosphorylation status, 2 d after TAC. As demonstrated in Fig. 6, α-MHC-MCM^+/−^ hearts exhibited significant increases in both SERCA2a mRNA (Fig. 6c; *P* < 0.05) and protein expression (Fig. 6i; *P* < 0.001) and decreased PLN mRNA expression (Fig. 6d; *P* < 0.001) and protein level (Fig. 6j; *p* < 0.05), and slightly decreased p-PLN T17 (Fig. 6k; *P* < 0.01), leading to increase in the ratios of SERCA2a to PLN (Fig. 6l; *P* < 0.01) and p-PLN T17/PLN (Fig. 6m; *P* < 0.05). These changes were reversed in Piezo1 KO TAC-operated hearts (Fig. 6). Together, these results suggest that Piezo1 expression in the setting of pressure overload leads to altered Ca^2+^ handling and changes in SERCA2a and PLN levels. In particular, the increased SERCA2a and reduced PLN levels maximize Ca^2+^ uptake into the sarcoplasmic reticulum, working to reduce cytosolic Ca^2+^. This is magnified by the increased proportion of pPLN/PLN after TAC as phosphorylation of PLN at the CaMKII-specific site, threonine 17, results in reduced inhibition of SERCA2a by PLN. α-MHC-MCM^+/−^ and WT hearts also exhibited significant increases in both NCX1 mRNA (*Slc8a1)* (Fig. 6e,f; both *P* < 0.01) and protein expression 2 d after TAC (Fig. 6n,o; both *P* < 0.01). In contrast, loss of Piezo1 in KO hearts abolished the increase in NCX1, a result not recapitulated in TRPM4 KO hearts (Fig. 6e,f,n,o), indicative of the fact that Piezo1 is upstream of NCX1 and mediates the response of NCX1 to pressure overload.

**Extended Data Fig. 7:**
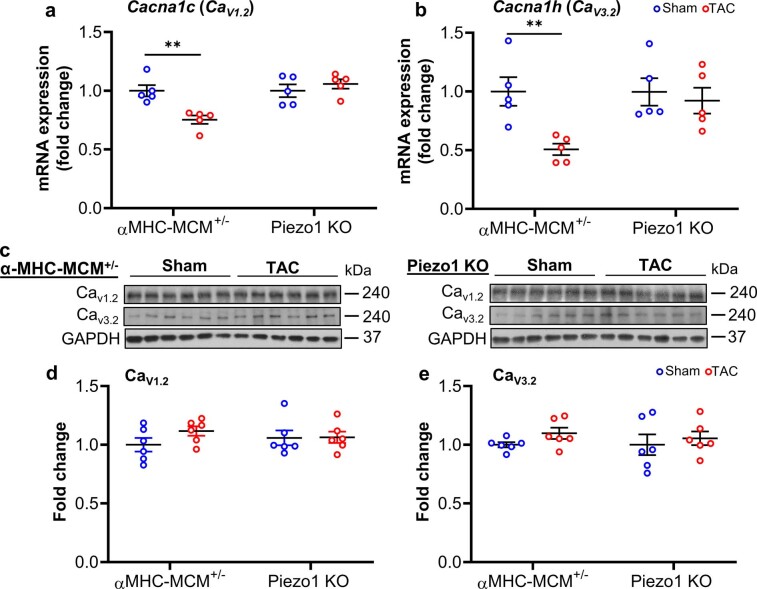
Gene expression and protein levels of *Cacna1c* (Ca_v1.2_) and *Cacna1h* (Ca_v3.2_) 2 days after TAC in Piezo1 KO and αMHC-MCM^+/-^ mouse hearts. (**a** and **b**) Relative mRNA expression of; (**a**) *Cacna1c (Ca*_*v1.2*_*)* and (**b**) *Cacna1h (Ca*_*v3.2*_*)* in LV tissue 2 days after sham or TAC. (**C**) Representative western blots of Ca_v1.2_ and Ca_v3.2_ in LV tissue. (**d**,**e**) Western blots from LV tissue after 2 days of TAC were used to quantify the protein levels of (**d**) Ca_v1.2_ and (**e**) Ca_v3.2_. Relative mRNA (n = 5/group) and protein expression (n = 6/group) in the LV tissue was normalized to GAPDH, respectively and mRNA expression was calculated as fold change relative to the 2 days post-sham group. Results are presented as mean ± SEM, two-way ANOVA with Tukey’s post-hoc test for multiple comparisons was used for **a**,**b** and Welch’s T-test, two-tailed for **d**,**e**, **p < 0.01 vs. sham-operated groups. Source data

We also looked at other potential modes of Ca^2+^ entry into cardiomyocytes. In particular, in α-MHC-MCM^+/−^ hearts, we observed significant decreases in the gene expression of the L-type (*Cacna1c*; Extended Data Fig. 7a; *P* < 0.01) and T-type Ca^2+^ channels (*Cacna1h*; Extended Data Fig. 7b; *P* < 0.01) 2 d after TAC (*P* < 0.01), which was abolished in Piezo1 KO hearts (Extended Data Fig. 7a,b), but the absence of any change at the protein level (Ca_v1.2_, Ca_v3.2_; Extended Data Fig. 7d,e) 2 d after TAC in α-MHC-MCM^+/−^ hearts casts some doubt on the functional significance of the L-type or T-type Ca^2+^ channels in mediating the response to pressure overload governed by Piezo1.

### Piezo1 and TRPM4 colocalize and physically interact

**Fig. 7:**
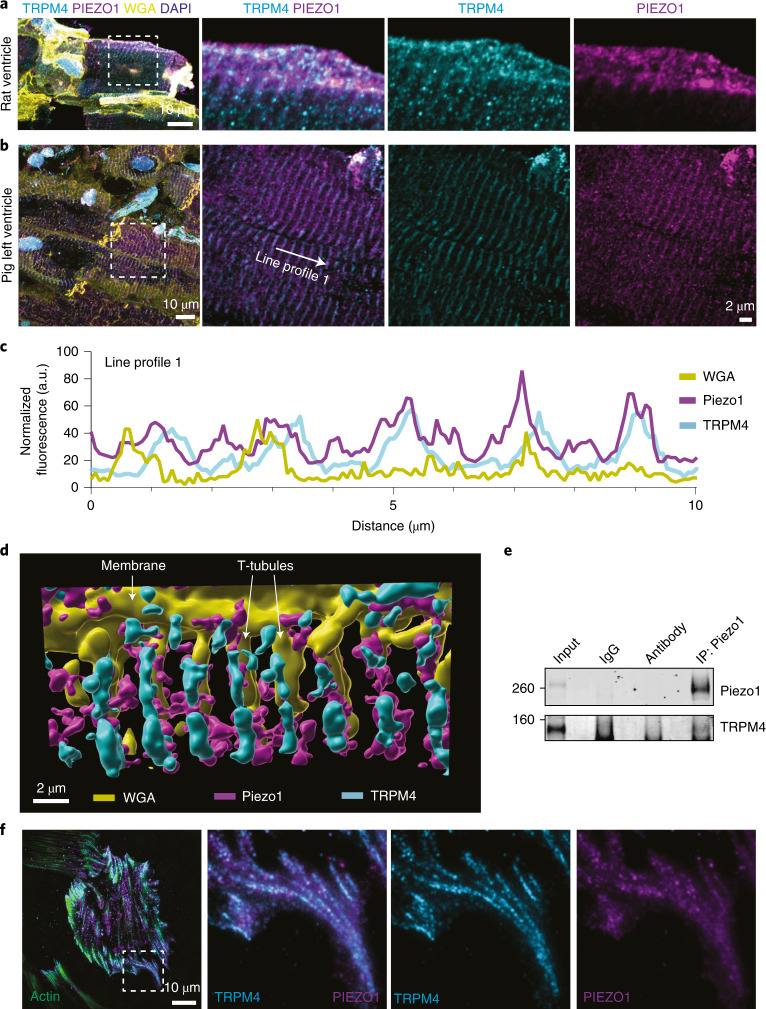
Colocalization and physical coupling of Piezo1 and TRPM4 in cardiomyocytes. **a**, Representative immunofluorescent images showing colocalization of TRPM4 and Piezo1 in rat ventricular myocytes; WGA, wheat germ agglutinin. **b**, Representative immunofluorescent images showing colocalization of TRPM4 and Piezo1 in pig ventricular myocardium. **c**, Representative line profile from **b** showing intensity of individual channels. **d**, Pseudo 3D reconstruction of confocal images from pig ventricular myocardium showing colocalization of Piezo1 and TRPM4 into T-tubules delineated with the membrane marker WGA. **e**, Immunoprecipitation of Piezo1 from H9c2 cardiomyocyte-like cells using an anti-Piezo1 antibody showing the co-IP of TRPM4. **f**, Representative total internal reflection fluorescence images showing the colocalization of Piezo1 and TRPM4 in H9c2 cells. a.u., arbitary fluorescent unit; IP, immunoprecipitate. Source data

To further explore the coupling of Piezo1 and TRPM4, we examined the colocalization of these channels. Due to the minimal tdTomato fluorescent signal in Piezo1–tdTomato mice and the lack of high-fidelity anti-mouse Piezo1 antibodies, we turned to LV tissue from rat and pig (Fig. 7). In both cases, we saw colocalization of Piezo1 and TRPM4, including overlap with wheat germ agglutinin (a plasma membrane marker) (Fig. 7a–c). This correlated with transverse tubules (T-tubules), a critical site for cardiomyocyte Ca^2+^ signaling. This is most notably seen with a pseudo three-dimensional (3D) reconstruction of confocal images showing the localization of both Piezo1 and TRPM4 along T-tubules (Fig. 7d). To further explore Piezo1/TRPM4 coupling, we utilized a cardiomyocyte cell line, rat H9c2 cells. While these cells do not replicate the structural environment of adult LV cardiomyocytes, they do provide a useful in vitro model. We first asked the question of whether these channels could be physically linked. To answer this, we carried out immunoprecipitation of Piezo1 in H9c2 cells and interrogated whether there was evidence that TRPM4 co-immunoprecipitated. Indeed, we found TRPM4 co-immunoprecipitation (co-IP) with Piezo1 compared with antibody-only and IgG controls (Fig. 7e). Finally, we confirmed the close proximity of Piezo1 and TRPM4 in H9c2 cells using total internal reflection fluorescence microscopy (Fig. 7f). This physical coupling maximizes the likelihood that the activation of Piezo1, which is expressed at low levels in cardiomyocytes, can stimulate TRPM4 to amplify the mechanical signal.

## Discussion

Recently, we demonstrated TRPM4 is a positive regulator of LVH induced by pressure overload, playing a key role in the activation of the CaMKII-HDAC4-MEF2 hypertrophic signaling pathway^7^. Activation of CaMKII and downstream hypertrophic signaling were inhibited after TAC in a mouse model in which TRPM4 was deleted in cardiomyocytes (TRPM4 KO), and this was associated with significant inhibition of the hypertrophic response to pressure overload. Since TRPM4 is not a stretch-activated^13^ channel but is Ca^2+^-activated, we postulated that TRPM4 must be downstream of a stretch-activated source of Ca^2+^. The primary aim of the current study, therefore, was to identify that stretch-activated Ca^2+^ source. Piezo1 was our prime candidate for this role because it is both stretch-activated^14,32,33^ and Ca^2+^-permeable (refs. ^14,34^). In addition, because CaMKII is a Ca^2+^/calmodulin-dependent kinase and TRPM4 is not Ca^2+^-permeable, it was apparent that TRPM4’s role in CaMKII activation must depend on one or more downstream sources of the Ca^2+^ that activates CaMKII via calmodulin.

This study identified the Ca^2+^-permeable Piezo1 mechanosensitive ion channel as the primary mechanotransducer that initiates the hypertrophic response to pressure overload via TRPM4 and the CaMKII-HDAC4-MEF2 signaling pathway. Using a conditional, cardiomyocyte-specific Piezo1 KO mouse model, we showed that deletion of Piezo1 completely prevented activation of the CaMKII-HDAC4-MEF2 hypertrophic signaling pathway after TAC, and this was associated with significant inhibition of the hypertrophic response to pressure overload. We then demonstrated in the Piezo1 KO mouse that Piezo1 was upstream of TRPM4 in the hypertrophic signaling cascade after TAC, and that Piezo1 controlled TRPM4 gene and protein expression early after TAC. We also showed in a TRPM4 KO mouse that TRPM4 contributed to the feedback mechanism that increased Piezo1 expression after TAC.

In the Piezo1 KO mouse, we also demonstrated that Piezo1 controls the expression of several Ca^2+^ handling proteins after TAC, most notably NCX1. Although a principal mechanism of cardiomyocyte Ca^2+^ extrusion, NCX dynamically changes the direction of ionic movement based on local ion concentrations and transmembrane voltage. NCX1 could explain increased intracellular Ca^2+^ concentration ([Ca^2+^]_i_), therefore, if the dynamic equilibrium shifted transiently in each beat to favor Ca^2+^ entry in response to high local intracellular Na^+^ concentration ([Na^+^]_i_). Given that TRPM4 is Na^+^-permeable, its activation by Piezo1 could account for the transient spike in [Na^+^]_i_ necessary to drive the NCX1 equilibrium transiently in favor of Ca^2+^ influx in exchange for Na^+^ efflux, which has been demonstrated elsewhere^35–39^.

**Fig. 8:**
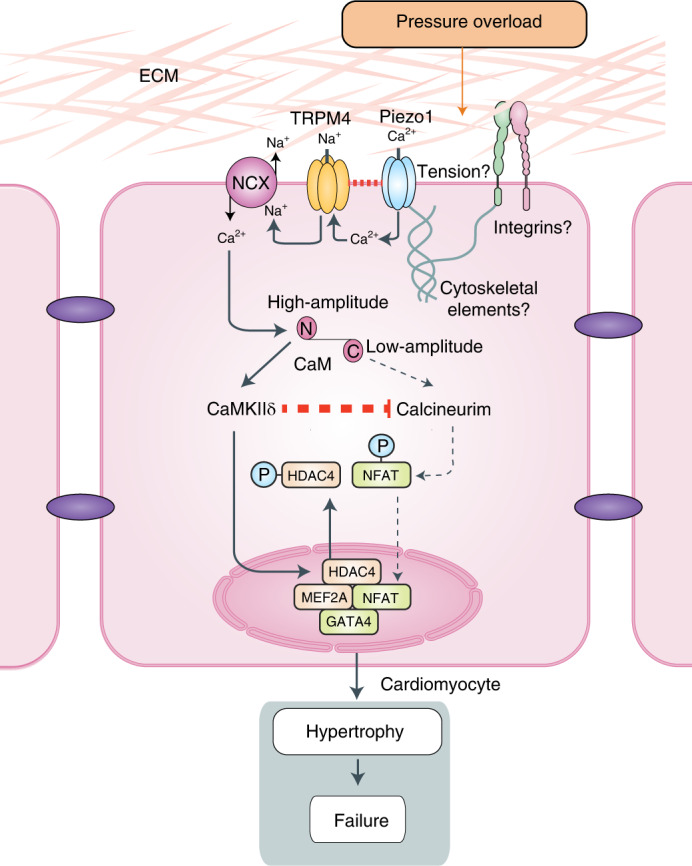
Schematic of the Piezo1-dependent signaling pathway that drives LVH secondary to pressure overload. Piezo1 is the cardiomyocyte mechanotransducer that converts the increased mechanical forces (either through membrane tension or force conveyed by cytoskeletal elements or integrins) secondary to pressure overload into Ca^2+^ entry into the cell. This increases local [Ca^2+^], resulting in TRPM4 activation. The Na^+^-permeable TRPM4 activity increases local [Na^+^], driving the NCX to extrude Na^+^, resulting in less extrusion of Ca^2+^ or Ca^2+^ entry via NCX. This leads to a high-amplitude increase in local [Ca^2+^]. Calmodulin responds to this high-amplitude Ca^2+^ stimulus through the lower-affinity Ca^2+^ binding site at its N-lobe, which then preferentially activates CaMKIIδ and thus stimulates the CaMKII-HDAC4-MEF2 pathway to induce LVH^6^. In contrast, calcineurin is activated preferentially by low-amplitude Ca^2+^ signaling via Gq-coupled receptors and calmodulin, and calcineurin activation is strongly inhibited by activated CaMKIIδ. These two mechanisms explain the strong functional segregation of the two hypertrophic signaling pathways despite their common dependence on activation via Ca^2+^/calmodulin. ECM, extracellular matrix; CaM, calmodulin.

These observations support the hypertrophic signaling pathway outlined schematically in Fig. 8. The first step in this pathway is the mechanical stretch activation of Piezo1 in cardiomyocytes by the increased myocardial forces associated with pressure overload. Stretch activation of Piezo1 in cardiomyocytes results in Ca^2+^ entry into the cell, increasing local [Ca^2+^]_i_ which then activates the Na^+^-permeable TRPM4 channel. The colocalization and physical interaction of these channels that we have identified across different organisms is likely to magnify this functional coupling. The resultant increase in local [Na^+^]_i_ postulated here would reduce Ca^2+^ extrusion via NCX1 at each beat and may also increase Ca^2+^ entry via NCX1 (ref. ^40^). This would amplify the increase in local [Ca^2+^]_i_ initiated by Piezo1. CaMKII is preferentially activated in response to high-frequency, high-amplitude Ca^2+^ oscillations^41^, and it is known that aortic constriction provides this type of Ca^2+^ signal^42^. In contrast, calcineurin activation requires a sustained increase in the resting intracellular Ca^2+^ level^43^. There is evidence that calmodulin can distinguish high-amplitude versus low-amplitude Ca^2+^ signals via the differential Ca^2+^ sensitivity of its N- and C-lobes^44,45^, and may thus provide a gatekeeper role in the differential activation of the CaMKII-dependent and calcineurin-dependent hypertrophic signaling pathways, with the high-amplitude Ca^2+^ stimulus activating calmodulin through the lower-affinity Ca^2+^ binding site at its N-lobe, followed by activation of the CaMKII-HDAC4-MEF2 pathway (Fig. 8). In addition, once activated, CaMKII inhibits calcineurin activation^8^.

Our study does not address explicitly the mechanism by which an increase in LVSP causes stretch activation of Piezo1, but there are several routes by which this could occur. First, Piezo1 can be activated by increased membrane tension^32,33^, which might reasonably be expected due to increased membrane forces associated with pressure overload. Force could also be transduced to the Piezo1 channel via cytoskeletal tethers or crosstalk with proteins that link the cardiomyocyte to the extracellular matrix, such as integrins, many of which have been implicated in the development of cardiac hypertrophy^46–48^. Our studies of ventricular myocytes from multiple organisms suggest localization of both Piezo1 and TRPM4 to the T-tubular system^19^. Membrane stretch in the T-tubular system under increased afterload could activate Piezo1 (ref. ^49^). Recent imaging clearly shows that the mechanical distortion of the T-tubular system of cardiomyocytes under load results in changes in caveolae density^50^, a cellular structure heavily influenced by membrane tension^51^. Systolic force generation during cardiomyocyte contraction stretches the T-tubules^49^, and an increase in afterload increases systolic force generation by the cardiomyocytes.

We reported previously that calcineurin activation was not required for the hypertrophic response to pressure overload with TAC^6^. In the present study, we found that cardiomyocyte-specific deletion of Piezo1 prevented activation of the CaMKII-HDAC4-MEF2 signaling pathway and reduced the amount of LVH developed after TAC by ~60%. While this observation strengthens the causal link between activation of the CaMKII-HDAC4-MEF2 signaling pathway and the induction of LVH secondary to pressure overload^8^, it might reasonably have been expected that the complete absence of CaMKII activation in Piezo1 KO hearts would prevent any hypertrophic response. Our findings support the conclusion, however, that the residual LVH in Piezo1 KO hearts after TAC may be due to more limited CaMKII-dependent inhibition of calcineurin allowing calcineurin-dependent NFAT translocation to the nucleus. We observed no evidence of calcineurin activation after TAC in the α-MHC-MCM^+/−^ (control) hearts, consistent with our previous report^6^, but in Piezo1 KO hearts in which CaMKII activation after TAC was abolished, calcineurin activation was clearly evident. It is notable, however, that calcineurin activation in Piezo1 KO hearts after TAC produced significantly less LVH than observed with CaMKII activation when Piezo1 was present and calcineurin activation was absent, emphasizing the importance of the CaMKII-HDAC4-MEF2 signaling pathway in pressure overload LVH.

In the same previous report^6^, we also demonstrated that Gq receptor activation was not required for the hypertrophic response to pressure overload with TAC since we found the same amount of LVH after TAC in the presence and absence of functioning Gq receptors. This raises the question: how is calcineurin activated after TAC in Piezo1 KO mice? It is important to note that TAC increases diacylglycerol, a downstream marker of Gq activation^52^. The implication of the present results, therefore, is that Gq is indeed activated directly by TAC, but that activation has no impact on the hypertrophic response in the presence of Piezo1 activation because Piezo1 activation results in CaMKII activation, which inhibits Gq-dependent calcineurin activation. When Piezo1 is deleted, CaMKII is not activated and Gq-dependent calcineurin activation in response to TAC is no longer inhibited. We observed the same phenomenon of calcineurin activation associated with residual LVH after TAC in the TRPM4 KO mouse, in which CaMKII activation was strongly inhibited^7^.

Although pressure overload resulted in a significant increase in *Piezo1* mRNA 2 d after TAC, Piezo1 protein was not increased at this early time point when evidence of hypertrophic signaling is already abundant. Since we have shown that Piezo1 is the instigator of the hypertrophic signaling cascade after TAC, it seems that the normal very low expression of Piezo1 protein is sufficient for this purpose due to the amplification of the Ca^2+^ signal by TRPM4. This raises an interesting question regarding the late increase in Piezo1 protein observed 14 d after TAC, a time when the hypertrophic response to TAC has already plateaued^6^. We speculate that stretch-activated Ca^2+^ entry via Piezo1 may be maximal when Piezo1 protein expression is low, and that increased Piezo1 protein production may act to reduce stretch activation and Ca^2+^ entry by cooperative load sharing between the increased Piezo1 molecules, and this is the subject of our ongoing research.

An independent research group has very recently reported inhibition of hypertrophy after TAC in a Piezo1 KO mouse model based on the same Cre-recombinase strategy used in our experiments^53^. While the inhibition of pressure overload hypertrophy with Piezo1 deletion is confirmatory, the authors did not examine the mechanism of the inhibition of hypertrophy in vivo and did not examine the CaMKII-HDAC4-MEF2 hypertrophic signaling pathway or the central role of TRPM4 in amplifying the initial Ca^2+^ signal provided by Piezo1. Based only on in vitro experiments in neonatal rat ventricular cardiomyocytes (NRVCMs) subjected to Yoda1, a specific activator of Piezo1 (ref. ^54^), the authors reported increased activity of calcineurin and calpain without increased expression of either protein. Stimulation of Piezo1 with Yoda1 produces a tonic increase in the Ca^2+^ concentration^54,55^, which is well known to activate calcineurin^43^, but this is not relevant to the high-amplitude Ca^2+^ signal produced by aortic constriction and necessary for CaMKII activation^41,42^. Given the differences also in maturity between NRVCMs and adult cardiomyocytes and the absence of data on CaMKII activation in their study of NRVCMs, it is difficult to determine the relevance of the NRVCM data to our findings in adult mice. We did demonstrate in adult mice in vivo, however, that due to inhibition of calcineurin activation by activated CaMKII, calcineurin activation is absent in the hypertrophic response to TAC initiated by Piezo1.

In summary, our study not only identifies Piezo1 as the primary instigator of hypertrophic signaling in response to pressure overload, but also provides a plausible mechanistic explanation for the central role of TRPM4 in amplifying the initial Ca^2+^ signal via NCX1 to activate CaMKII via calmodulin, and thus activate the CaMKII-HDAC4-MEF2 hypertrophic signaling pathway. In addition, our findings reveal the potency of the inhibition of calcineurin activation by activated CaMKII as the main explanation for the apparent segregation of the CaMKII-mediated and calcineurin-mediated hypertrophic signaling pathways, despite the fact that activation of both CaMKII and calcineurin is Ca^2+^/calmodulin-dependent. The proximity and physical link we have demonstrated between Piezo1 and TRPM4 suggest that their stretch-activated functional coupling could provide a universal paradigm for Piezo1’s broader role in all biological tissues. These findings may also provide targets for the development of therapies to prevent or reverse pathological hypertrophy and its harmful clinical sequelae.

## Methods

### Mice and genotyping

All experimental procedures were approved by the Animal Ethics Committee of Garvan/St Vincent’s (Australia), in accordance with the guidelines of both the Australian Code for the care and use of animals for scientific purposes (8th edition, National Health and Medical Research Council, Australia, 2013) and the Guide for the Care and Use of Laboratory Animals (8th edition, National Research Council, USA, 2011). The mice were maintained in a light/dark cycle of 12 h/12 h, at a temperature of 21 °C and 50% humidity.

The homozygous Piezo1 reporter mice expressing a fusion protein of Piezo1 and the fluorophore tdTomato (*Piezo1*^*P1-tdT/P1-tdT*^; Jackson Laboratory, stock No. 029214) were backcrossed to C57BL/6J mice to yield heterozygous *Piezo1*^*P1-tdT/wt*^ mice that were intercrossed to each other to obtain homozygous *Piezo1*^*P1-tdT/P1-tdT*^ mice for the experiments, and their WTLs served as controls.

To generate inducible cardiomyocyte-specific Piezo1 KO mice, we crossed homozygous Piezo1^flox/flox^ mice (Jackson Laboratory, stock No. 029213) with homozygous *Myh6*-MerCreMer mice (MCM), which have a tamoxifen-inducible Cre-recombinase under the control of the αMHC (*Myh6)* promoter^20^, to produce Piezo1^flox/flox^;αMHC-MCM^+/−^ (termed P1^fl/fl^MCM^+/−^) mice. The age- and sex-matched Piezo1^wt/wt^;αMHC-MCM^+/−^ (termed αMHC-MCM^+/−^) mice and Piezo1^wt/wt^;αMHC-MCM^−/−^ (termed P1^wt/wt^MCM^−/−^) mice were used as controls for experiments characterizing phenotype at baseline.

To induce Cre-recombinase-mediated deletion of exons 20–23 of the Piezo1 gene selectively in cardiomyocytes of adult mice, a daily intraperitoneal injection of tamoxifen (30 mg kg^−1^; Sigma, T5648) dissolved in 95% peanut oil (Sigma, P2144) was administered for 6 consecutive days to male P1^fl/fl^MCM^+/−^ mice aged 8–10 weeks (termed Piezo1 KO). The age- and sex-matched P1^wt/wt^αMHC-MCM^+/−^ mice treated with tamoxifen served as Cre-only controls. P1^fl/fl^MCM^+/−^ mice treated with peanut oil acted as flox controls. Mice were given 10 d to recover after receiving the last injection of tamoxifen before experiments. Mice were genotyped using the following primers: P1 F: CTT GAC CTG TCC CCT TCC CCA TCA AG; P1 WT/fl R: CAG TCA CTG CTC TTA ACC ATT GAG CCA TCT C; P1 KO R: AGG TTG CAG GGT GGC ATG GCT CTT TTT using Phire II polymerase (Thermo Scientific) using the following cycling conditions: initial denaturation 98 °C for 30 s; followed by 31 cycles of 98 °C for 5 s, 65 °C for 5 s, 72 °C for 5 s; followed by a final hold of 72 °C for 2 min. Reactions were separated on 2% agarose gels yielding the following band sizes: P1^+^: 160 bp, P1^f^: 330 bp, P1^−^: 230 bp (ref. ^23^). All mice were on the C57BL/6J genetic background, and male mice aged 8–13 weeks were used throughout.

### Induction of LVH

As previously described^6,7^, male mice were subjected to TAC to induce pressure overload. Mice were anesthetized with 5% isoflurane and ventilated at 120 breaths per min (Harvard Apparatus Rodent Ventilator). The transverse aortic arch was accessed via an incision in the second intercostal space and constricted with a ligature tied around a 25-gauge needle, which was then removed. The TAC procedure was modified from a published paper^56^. Sham mice underwent the same procedure, but the ligature was not tied. Simultaneous direct pressure recordings (1.4 F (French catheter scale) pressure catheter, AD Instruments) from both the right carotid artery and the aorta distal to the ligature (*n* = 20 mice) indicated a TAC pressure gradient of 60 ± 8 mmHg with this technique. Animals were killed after 2 d or 14 d of surgery.

### Echocardiographic measurements

As previously described^57^, echocardiography was performed using an MS400 18–38 MHz transducer probe and VEVO 2100 ultrasound system (VisualSonics). The mice were anesthetized (3–5% isoflurane for induction, 1–2% isoflurane for maintenance with adjustment to maintain heart rate at ~500  beats per minute [bpm]) and imaged at the endpoint of the study to assess cardiac function. The acquisition of images and evaluation of data were performed by an operator blinded to treatment.

### Invasive hemodynamic measurements

As previously described^6,7^, after 14 d of sham or TAC, mice were anesthetized by inhalation of isoflurane (1.5%) and a 1.4 F micro-tip pressure catheter (Millar Instruments) was inserted into the left ventricle via the right carotid artery. The heart rate, aortic systolic pressure, LVSP, +d*P*/d*t* and −d*P*/d*t* were recorded (LabChart 6 Reader, AD Instruments). Animals were killed, and the heart weight and LVW normalized to body weight and to tibia length were measured as indicators of LVH.

### Mouse LV cardiomyocyte isolation and purification

As previously described^7^, the mice were heparinized and euthanized according to the Animal Research Act 1985 No. 123 (New South Wales, Australia). Hearts were dissected and perfused through the aorta and the coronary arteries by 10 ml of pH 7.2 perfusion buffer containing 135 mM NaCl, 4 mM KCl, 1 mM MgCl_2_, 0.33 mM NaH_2_PO_2_, 10 mM HEPES, 10 mM glucose, 10 mM 2,3-butanedione 2-monoxime (BDM) and 5 mM taurine, with a Langendorff apparatus at 37 °C for 5 min. Next, 30 ml of digestion buffer composed of the above solution and Collagenase B and D (dose by body weight: 0.4 mg g^−1^, Roche) and Protease Enzyme Type XIV (dose by body weight: 0.07 mg g^−1^, Sigma-Aldrich) was used to perfuse the hearts for 15 min. After the perfusion, the heart was removed from the setup and placed into a pH 7.4 transfer buffer (Buffer A) containing 135 mM NaCl, 4 mM KCl, 1 mM MgCl_2_, 0.33 mM NaH_2_PO_2_, 10 mM HEPES, 5.5 mM glucose, 10 mM BDM and 5 mg ml^−1^ BSA. Both atria and the right ventricle were discarded, and the LV muscle was torn into small pieces and gently triturated in transfer buffer with a pipette to isolate cardiomyocytes. The suspension was then filtered through a 200-μm Falcon cup filter (BD) and centrifuged at 20*g* for 2 min. After that, the cardiomyocytes were used either for Ca^2+^ imaging experiments or for purification described previously^58,59^, which confirmed that rod-shaped cardiomyocytes accounted for more than 85% of the total purified cardiomyocytes. The isolated cardiomyocytes were frozen immediately in liquid nitrogen and stored at −80 °C for the following experiments.

### Cardiomyocyte Ca^2+^ imaging

For Ca^2+^ imaging experiments, another pH 7.4 transfer buffer (Buffer B) which contained 135 mM NaCl, 5.4 mM KCl, 0.5 mM MgCl_2_, 1.8 mM CaCl_2_, 10 mM HEPES, 5.5 mM glucose, 10 mM BDM and 5 mg ml^−1^ BSA was prepared. Buffer A and Buffer B were mixed in different ratios to make solutions with Ca^2+^ concentrations of 0.06, 0.24, 0.6 and 0.8 mM. To avoid intracellular Ca^2+^ overload, isolated mouse LV cardiomyocytes were transferred sequentially into the solutions to gradually adapt to a final Ca^2+^ concentration of 0.8 mM. Then the cells were seeded into a 96-well cell culture microplate (Greiner Bio-One) at 1,500 cells per well, and incubated with fluorescent Ca^2+^ indicator 2.5 µM Cal-520, AM (Abcam) at room temperature for 40 min. Subsequently, the cells were rinsed with and maintained in 100 μl of Buffer A and B mixture (0.8 mM Ca^2+^). Ca^2+^ imaging and data recording were carried out on a Nikon Eclipse Ti2-E Inverted epifluorescence microscope (Nikon Instruments), using a ×20 objective lens, with 2 × 2 binning imaging at 50 frames per s. The data were continuously recorded for 160 s in total. At 40 s of each recording a final concentration of 30 μM of Yoda1 was added to each well. This is in addition to a large cohort of cells being imaged in the absence of Yoda1. NIS-Elements Microscope Imaging software, v.5.11.03 (Nikon Instruments), was used for Ca^2+^ imaging data analysis.

### Quantitative polymerase chain reaction (qPCR)

Gene expression was determined by qPCR. Total RNA was extracted and purified from LV tissue and isolated cardiomyocytes with the RNeasy Fibrous Tissue Mini Kit (QIAGEN), following the manufacturer’s protocol. RNA (500 ng) was reverse transcribed into complementary DNA using the SuperScript III First-Strand Synthesis SuperMix kit (Invitrogen). cDNA was subjected to PCR amplification to detect ANP (*Nppa*), BNP (*Nppa*), α-SA (*Acta1*), collagen III (*Col3a1*), *Piezo1*, *Trpm4*, *SERCA2a*, PLN (*PLN*), *Slc8a1*, *Cacna1c* (*Ca*_*v1.2*_) and *Cacna1h* (*Ca*_*v3.2*_) gene expression, using the PCR master mix LightCycler 480 SYBR Green I Master (Invitrogen) and performed with the CFX384 Touch Real-Time PCR Detection System (Bio-Rad). Samples were run in technical triplicates and the mRNA expression levels were normalized to those of GAPDH to calculate relative gene expression using the delta-delta C_t_ method. The mouse qPCR primers (Sigma-Aldrich) used are shown in Supplementary Table 3.

### Western blotting

For total protein extraction, LV tissue and isolated cardiomyocytes were lysed in a pH 7.4 lysis buffer containing 150 mM NaCl, 50 mM Tris-HCL, 1% Triton X-100, 1 mM sodium orthovanadate, 1 mM beta-glycerophosphate, 5 mM dithiothreitol and MiniComplete protease inhibitors (Roche). The cytoplasmic and the nuclear fractions of LV tissue were separated as described previously with confirmed high fraction purity^6,7^. Briefly, LV tissue was lysed using NE-PER nuclear and cytoplasmic extraction reagents (Pierce Biotechnology) and Protease Inhibitor Cocktail Kit and Halt Phosphatase Inhibitor Cocktail (Pierce Biotechnology), both with a homogenizer (PRO Scientific). Protein (30 μg for each sample) was loaded on 4–20% Mini-PROTEAN TGX Gels (Bio-Rad) and separated by electrophoresis. Samples were transferred to PVDF membranes (Bio-Rad), blocked with 5% BSA, then labeled overnight with primary antibodies, except for anti-SERCA2a for 1 h at 4 °C (Supplementary Table 3): anti-mCherry (1:500; Thermo Scientific), anti-SERCA2a (1:35,000; Abcam), anti-Phospholamban (1:1,500; Abcam), anti-Phospho-Phospholamban (Thr17, 1:1,500; Badrilla), anti-CACNA1C (Ca_v1.2_, 1:10,000; Abcam), anti-CACNA1H (Ca_v3.2_,1:2,000; Abcam), anti-NCX1 (1:1,000; Thermo Scientific), anti-TRPM4 (1:200; Alomone Labs), anti-CaMKIIδ (1:1,000; Abcam), anti-p-CaMKII (Thr287, 1:5,000; Thermo Scientific), anti-HDAC4 (1:1,500; Cell Signaling), anti-p-HDAC4 (Ser632, 1:1500; Cell Signaling), anti-MEF2A (1:3,000; Cell Signaling), anti-NFATc4 (1:1,500; Abcam) and anti-GATA4 (1:1,000; Santa Cruz Biotechnology). Anti-GAPDH (1:10,000; Cell Signaling Technology) and anti-Histone H2B (1:5,000; Abcam) were used to standardize sample loading. Horseradish peroxidase (HRP)-conjugated goat anti-rabbit (1:10,000) or rabbit anti-mouse (1:5,000) or goat anti-rat (1:5,000) secondary antibodies (Abcam) (Supplementary Table 3) were used at room temperature for 1 h. Immunologic detection was accomplished using SuperSignal West Pico PLUS Chemiluminescent Substrate (Thermo Scientific). Uncropped images of blots are provided as source data. Protein levels were quantified by densitometry using ImageJ (NIH) software, v.1.52p. Protein levels were normalized to relative changes in Histone H2B for the nuclear fraction and GAPDH for the cytoplasmic fraction, which were run in parallel on different gels at the same time using the same experimental samples and expressed as fold changes relative to those of control animals.

### Histological and immunofluorescence analyses

As previously described^7^, Masson’s trichrome stain was used to quantify fibrosis in the LV (collagen fibers stain blue). The hearts were excised from isoflurane-euthanized mice and washed with PBS. Hearts were then cut longitudinally in the coronal plane, embedded into optimal cutting temperature (OCT) compound (Sakura Finetek) and gradually frozen in melting isopentate, precooled in liquid nitrogen to avoid tissue damage and stored at −80 °C for the following experiments. Serial 6-μm sections were sliced with a cryostat (Leica) and stained using a Masson’s trichrome staining kit (Sigma-Aldrich), following the manufacturer’s instructions. LV images were obtained with 4–6 fields per section^60^ using a brightfield microscope (Leica). Blue-stained areas of fibrosis within sections were determined using color-based thresholding^61^ and measured with ImageJ software (NIH; http://rsbweb.nih.gov/ij/). The percentage of total fibrotic area was calculated by taking the sum of the blue-stained areas and dividing by the total LV area.

Whole pig hearts were collected from a local abattoir (Picton MeatWorx) in Krebs buffer, on ice. Ventricular tissues were dissected, embedded in OCT and processed for immunolabelling and confocal imaging as described above. Line profile intensity data were generated using the ZEN Blue software (ZEISS).

Paraffin-embedded rat heart sections were a kind gift from Prof. Peter MacDonald (Victor Chang Cardiac Research Institute [VCCRI]/St Vincent’s Hospital). Following standard deparaffination, sections were subjected to antigen retrieval using 0.05% Trypsin at 37 °C for 20 min before immunolabelling and confocal imaging as described above.

The H9c2 cells were a kind gift from Prof. Richard Harvey (VCCRI). The cells were maintained in DMEM (Sigma-Aldrich) + 20% FBS (HyClone, GE Healthcare Life Sciences) at 37 °C with 5% CO_2_. Single cells were plated overnight on No. 1.5 glass coverslips. The next day, they were fixed with 4% paraformaldehyde in PBS for 20 min at room temperature, quenched with 0.1 M glycine for 10 min, then permeabilized with 0.05% saponin for 10 min. Primary antibodies were diluted in 1% BSA + 0.005% saponin and incubated for 1 h at room temperature, followed by the appropriate secondary antibodies diluted in the same buffer for 1 h at room temperature. Finally, actin fibers were stained with phalloidin-FITC, membranes were labeled with Wheat Germ Agglutinin and nuclei were labeled with DAPI. The samples were imaged in PBS using total internal reflection fluorescence microscopy (Elyra7, ZEISS, ×63 objective). 3D surface rendering was performed using Imaris (Imaris X64, v.9.5.1).

Primary antibodies used were a rabbit polyclonal anti-TRPM4 (NBP2-13487, Novus Biosciences), a mouse monoclonal anti-Piezo1 (NBP2-76517, Novus Biosciences), a rat monoclonal anti-beta1 integrin (550531, BD Biosciences), a rat monoclonal anti-CD31 (550274, BD Biosciences) and a rabbit polyclonal anti-RFP (600-401-379, Rockland). Secondary antibodies used were a goat anti-rat conjugated to AlexaFluor488 (A11006, Invitrogen), a goat anti-mouse conjugated to AlexFluor647 (ab150119, Abcam), a donkey anti-rabbit conjugated to CF640 (20178, Biotium) and a donkey anti-mouse conjugated to AlexaFluor555 (A31570, Invitrogen).

### Co-IP assay

As previously described^62^, H9c2 cells were lysed with a co-IP buffer containing 25 mM NaPIPES (pH 7.2), 140 mM NaCl, 1 mM EDTA, 1% CHAPS, 0.5% phosphatidylcholine, 2 mM dithiothreitol and a cocktail of protease inhibitors (Roche) on ice for 30 min. After centrifugation at 4 °C, 15,000*g* for 15 min, the supernatant was incubated with protein G Dynabeads (Thermo Fisher) for 2 h at 4 °C (pre-clear). After pre-clearing, the mixture was centrifuged again, as above, and the supernatant was incubated with either rabbit IgG or anti-Piezo1 antibody at 4 °C overnight. The beads were then washed three times with a washing buffer containing 25 mM NaPIPES (pH 7.2), 140 mM NaCl, 0.6% CHAPS, 0.14% phosphatidylcholine, 2 mM dithiothreitol and a cocktail of protease inhibitors, and finally heated at 62 °C for 5 min with 2× SDS protein loading buffer. The elution was subjected to SDS–PAGE and western blotting. Anti-TRPM4 (1:200; Alomone Labs) and anti-Piezo1 (immunoprecipitation: 0.85 μg; western blot: 1:1,000; Alomone Labs) were used in the experiments.

### Statistics

All experiments and analyses were blinded. Statistics used for every figure or table are indicated in the corresponding figure or table legend. Averaged data are presented as mean ± s.e.m. The statistical analyses were performed using GraphPad Prism software, v.7.04 (GraphPad). For comparisons among three or more sets of data with one factor or two factors, one-way or two-way analysis of variance (ANOVA) was used, accordingly, followed by Tukey’s post hoc test. For comparisons between two groups, Welch’s *t*-test, two-tailed, was used. *P* < 0.05 was considered statistically significant. All samples used in this study were biological replicates, not technical replicates. All experiments were conducted using at least two independent materials to reproduce similar results.

### Reporting summary

Further information on research design is available in the Nature Research Reporting Summary linked to this article.

## Supplementary Information

Supplementary Information Supplementary Tables 1–3, Figs. 1 and 2 and Additional Figs. 1 and 2.

Reporting Summary

Supplementary Data Statistical source data for Supplementary Fig. 2 and Supplementary Tables 1 and 2.

## Source data

Source Data Fig. 1 Statistical source data.

Source Data Fig. 1 Full-length, unprocessed blots.

Source Data Fig. 2 Statistical source data.

Source Data Fig. 3 Statistical source data.

Source Data Fig. 4 Statistical source data.

Source Data Fig. 4 Full-length, unprocessed blots.

Source Data Fig. 5 Statistical source data.

Source Data Fig. 5 Full-length, unprocessed blots.

Source Data Fig. 6 Statistical source data.

Source Data Fig. 6 Full-length, unprocessed blots.

Source Data Fig. 7 Full-length, unprocessed blots.

Source Data Extended Data Fig. 2 Statistical source data.

Source Data Extended Data Fig. 3 Statistical source data.

Source Data Extended Data Fig. 4 Statistical source data.

Source Data Extended Data Fig. 5 Statistical source data.

Source Data Extended Data Fig. 6 Statistical source data.

Source Data Extended Data Fig. 7 Statistical source data.

Source Data Extended Data Fig. 7 Full-length, unprocessed blots.

Source Data Extended Data Table. 1 Statistical source data.

Source Data Extended Data Table. 2 Statistical source data.

Source Data Extended Data Table. 3 Statistical source data.

## Figures and Tables

**Extended Data Table 1:**
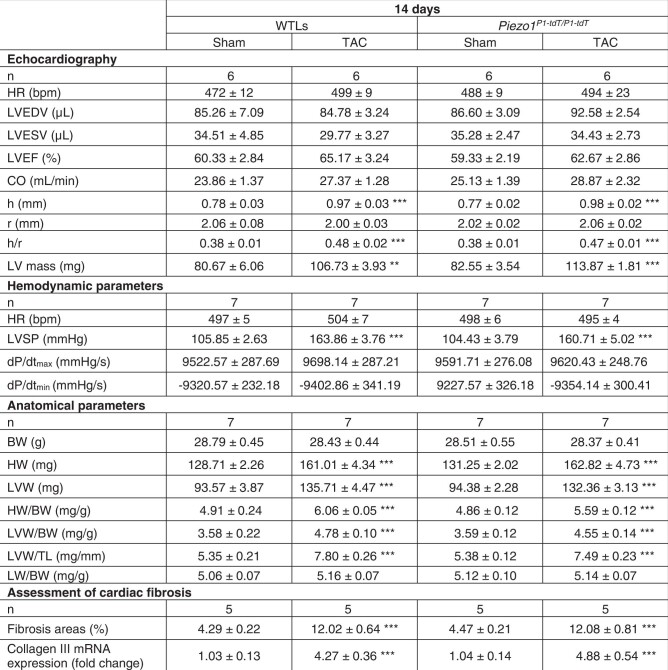
Echocardiographic, hemodynamic and anatomical parameters 14 days after TAC or sham surgery in WTLs and *Piezo1*^*P1-tdT/P1-tdT*^mice. Echocardiographic and hemodynamic parameters were measured in *Piezo1*^*P1-tdT/P1-tdT*^ mice and their WTLs 14 days after TAC or sham surgery (n = 6–7/group). Post-mortem analysis was performed 14 days after TAC or sham surgery. Cardiac fibrosis was evaluated by Masson’s trichrome staining, and the degree of fibrosis was graded (n = 5/group). Relative Collagen III (*Col3a1*) mRNA expression was normalized with GAPDH and calculated as fold change relative to the sham group, (n = 5/group). HR: heart rate; LVEDV: left ventricular (LV) end-diastolic volume; LVDSV: LV end-systolic volume; LVEF: LV ejection fraction; CO: cardiac output; h: LV wall thickness; r; chamber radius; h/r: LV wall thickness to chamber radius ratio. LVSP: left ventricular systolic pressure; HR: heart rate; dP/dt_max_: the peak rate of pressure increase; dP/dt_min_: the peak rate of pressure decrease; BW: body weight; HW: heart weight; LVW: left ventricular weight; LW: HW/BW: heart weight to body weight ratio; LVW/BW: LV weight to body weight ratio; LVW/TL: LV weight to tibia length ratio; LW/BW: lung weight to body weight ratio. Data are presented as means ± SEM, two-way ANOVA with Tukey’s post-hoc test for multiple comparisons was used to assess effects of genotype, surgery, and genotype by surgery interaction, **p < 0.01, ***p < 0.001 vs sham-operated groups for each respective genotype Source data

**Extended Data Table 2:**
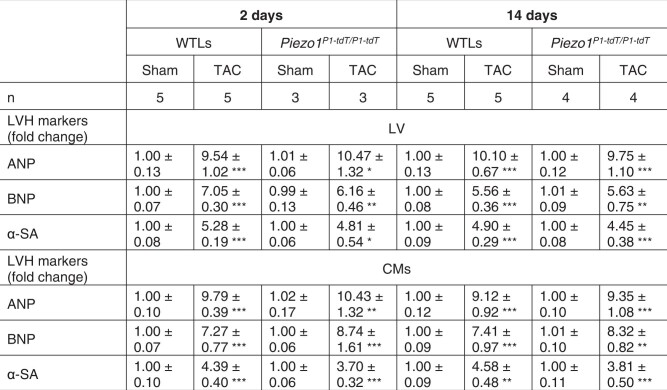
Early markers of LVH induction in response to left ventricular pressure overload in WTLs and *Piezo1*^*P1-tdT/P1-tdT*^ mice. Relative mRNA expression of ANP (*Nppa*), BNP (*Nppb*) and α-SA (*Acta1*) in LV tissue and cardiomyocytes (CMs) 2 days and 14 days after TAC compared to sham (n = 3–5/group). The relative mRNA expression was normalized with GAPDH and calculated as fold change relative to sham in 2 days and 14 days groups, respectively. Results are presented as mean ± SEM, two-way ANOVA with Tukey’s post-hoc test for multiple comparisons was used to assess effects of genotype, surgery, and genotype by surgery interaction *p < 0.05, **p < 0.01, ***p < 0.001 vs sham-operated groups for the respective genotype Source data

**Extended Data Table 3:**
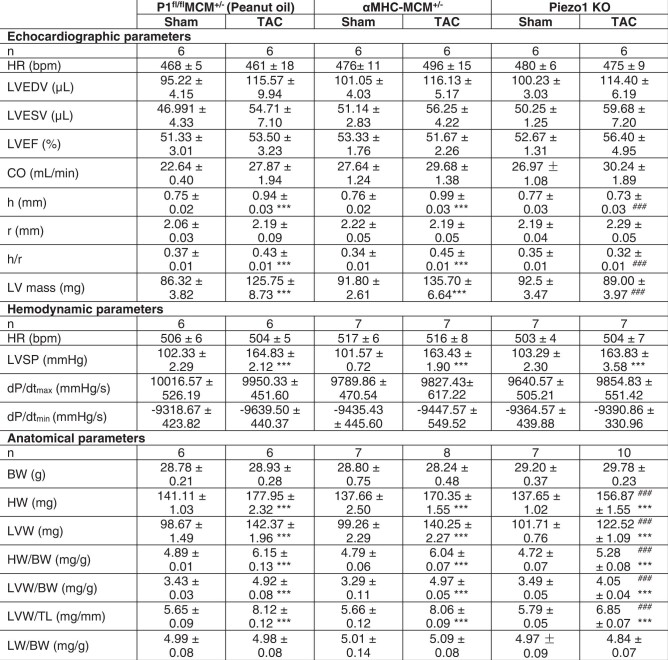
Echocardiographic, hemodynamic and anatomical parameters 14 days after TAC or sham surgery in αMHC-MCM^+/-^ mice and Piezo1 KO mice, and P1^fl/fl^MCM^+/-^ controls. Echocardiographic and hemodynamic parameters were measured in αMHC-MCM^+/-^ mice and Piezo1 KO mice, and P1^fl/fl^MCM^+/-^ controls 14 days after TAC or sham surgery (n = 6–7/group). Post-mortem analysis of mice was performed 14 days after TAC or sham surgery (n = 6–10/group). HR: heart rate; LVEDV: left ventricular (LV) end-diastolic volume; LVESV: LV end-systolic volume; LVEF: LV ejection fraction; CO: cardiac output; h: LV wall thickness; r; chamber radius; h/r: LV wall thickness to chamber radius ratio. LVSP: Left ventricular systolic pressure; HR: heart rate; dP/dt_max_: the peak rate of pressure increase; dP/dt_min_: the peak rate of pressure decrease; BW: body weight; HW: heart weight; LVW: left ventricular weight; LW: HW/BW: heart weight to body weight ratio; LVW/BW: LV weight to body weight ratio; LVW/TL: LV weight to tibia length ratio; LW/BW: lung weight to body weight ratio. Data are presented as mean ± SEM, two-way ANOVA with Tukey’s post-hoc test for multiple comparisons was used to assess effects of genotype, surgery, and genotype by surgery interaction, ***p < 0.001 vs sham-operated groups, ###*p* < 0.001 vs TAC- operated αMHC-MCM^+/-^ and -P1^fl/fl^MCM^+/-^ mice Source data

## Data Availability

Source data are provided with this article.
