# Supplementary material for: Piezo1 is the cardiac mechanosensor that initiates the cardiomyocyte hypertrophic response to pressure overload in adult mice
Source: Nat Cardiovasc Res. 2022 Jun 13;1(6):577–91. doi: 10.1038/s44161-022-00082-0 (PMC11358016; doi:10.1038/s44161-022-00082-0)
Supplement: Supplementary file 1 — Supplementary Tables 1–3, Figs. 1 and 2 and Additional Figs. 1 and 2. [file 44161_2022_82_MOESM1_ESM.pdf]

nature portfolio

https://doi.org/10.1038/s44161-022-00082-0

Supplementary information

Piezo1 is the cardiac mechanosensor that initiates the cardiomyocyte hypertrophic response to pressure overload in adult mice

In the format provided by the authors and unedited

Supplementary Information

Piezo1 is the cardiac mechanosensor that initiates the cardiomyocyte hypertrophic response to pressure overload in adult mice

Ze-Yan Yu[1,2,3], Hutao Gong[1,2,3], Scott Kesteven[2,3], Yang Guo[1,2,3], Jianxin Wu[1], Jinyuan Vero Li[1], Delfine Cheng[1,3], Zijing Zhou[1], Siiri E. Iismaa[1,3], Xenia Kaidonis[1], Robert M. Graham[1,3], Charles D. Cox[1,3], Michael P. Feneley[2,3,4#] & Boris Martinac[1,3#]

[1] Molecular Cardiology and Biophysics Division, Victor Chang Cardiac Research Institute, Sydney, New South Wales, Australia.

[2] Cardiac Physiology and Transplantation Division, Victor Chang Cardiac Research Institute, Sydney, New South Wales, Australia.

[3] St Vincent's Clinical School, Faculty of Medicine, University of New South Wales, Sydney, New South Wales, Australia.

[4] Department of Cardiology, St Vincent's Hospital, Sydney, New South Wales, Australia.

#To whom correspondence should be addressed:

Dr Boris Martinac

E-mail: b.martinac@victorchang.edu.au

Tel: (+61) 92958743

Dr Michael P. Feneley

E-mail: Michael.feneley@svha.org.au

Tel: (+61) 92958743

Supplementary Table 1. Anatomical parameters at 2 days after sham or TAC in WTLs and *Piezo1*$^{P1\text{-}tdT/P1\text{-}tdT}$ mice. Post-mortem analysis of parameters was measured in *Piezo1*$^{P1\text{-}tdT/P1\text{-}tdT}$ and their wild type littermates (WTLs) 2 days after being subjected to TAC or sham surgery (n = 6-7/group). BW: body weight; HW: heart weight; LVW: left ventricular weight; LW: HW/BW: heart weight to body weight ratio; LVW/BW: LV weight to body weight ratio; LVW/TL: LV weight to tibia length ratio; LW/BW: lung weight to body weight ratio. Data are presented as mean ± SEM, two-way ANOVA with Tukey's post-hoc test for multiple comparisons was used to assess effects of genotype, surgery, and genotype by surgery interaction.

	**2 days**			
	**WTLs**		***Piezo1*$^{P1\text{-}tdT/P1\text{-}tdT}$**	
	**Sham**	**TAC**	**Sham**	**TAC**
**Anatomical parameter**				
n	7	6	7	7
BW (g)	29.76 ± 0.39	29.27 ± 0.58	29.50 ± 0.48	28.57 ± 0.29
HW (mg)	132.71 ± 2.84	131.86 ± 2.59	135.17 ± 4.59	132.43 ± 2.58
LVW (mg)	98.57 ± 3.99	96.14 ± 3.97	97.33 ± 4.13	97.86 ± 3.22
HW/BW (mg/g)	4.45 ± 0.09	4.52 ± 0.08	4.54 ± 0.10	4.60 ± 0.12
LVW/BW (mg/g)	3.27 ± 0.10	3.34 ± 0.12	3.28 ± 0.08	3.44 ± 0.12
LVW/TL (mg/mm)	5.66 ± 0.22	5.51 ± 0.23	5,.60 ± 0.23	5.64 ± 0.19
LW/BW (mg/g)	4.91 ± 0.08	5.01 ± 0.09	4.96 ± 0.08	5.07 ± 0.04

Supplementary Table 2: Tamoxifen injection causes dose-dependent left ventricular dilatation and impaired contraction. Left ventricular function, wall thickness and mass were assessed by echocardiography 10 days after the last injection of tamoxifen at 30, 50 or 100 mg/kg/d on three consecutive days in $P1^{fl/fl}MCM^{+/-}$ and $^{P1wt/wt}MCM^{+/-}$ mice. $P1^{fl/fl}MCM^{+/-}$ mice injected with peanut oil provided control measurements. BW, body weight; HR, heart rate; LVEDV, LV end-diastolic volume; LVESV, LV end-systolic volume; LVEF, LV ejection fraction; CO, cardiac output; h, LV wall thickness; r, chamber radius; h/r ratio, LV wall thickness to chamber radius ratio and LV mass, n = 4/group. Results are presented as mean ± SEM, one-way ANOVA with Tukey's post-hoc test for multiple comparisons were used, *p < 0.05, **p < 0.01, ***p < 0.001 when compared with peanut oil injected group.

**Genotype**	**$P1^{fl/fl}MCM^{+/-}$**	**$P1^{fl/fl}MCM^{+/-}$**	**$P1^{fl/fl}MCM^{+/-}$**	**αMHC-$MCM^{+/-}$**	**$P1^{fl/fl}MCM^{+/-}$**	**αMHC-$MCM^{+/-}$**
**Treatment**	**Peanut oil (control)**	**Tamoxifen 30 mg**	**Tamoxifen 50 mg**	**Tamoxifen 50 mg**	**Tamoxifen 100 mg**	**Tamoxifen 100 mg**
n	4	4	4	4	4	4
**Parameters**						
BW (g)	28.5 ±0.5	28.6 ±0.4	29.5 ±1.4	28.4 ±1.0	29.2 ±1.3	30.1 ±1.1
HR (bpm)	477 ±15	489 ±44	463 ±17	438±18	385 ±16 *	384 ±9 *
LVEDV (µL)	82.3 ±3.9	77.2 ±2.9	138±7.2 ***	133 ±5.7 **	141 ±8.7 ***	146 ±13 ***
LVESV (µL)	29.3 ±3.5	27.9 ±1.2	114 ±6.7 ***	117 ±5.1 ***	120 ±7 ***	124 ±7.8 ***
LVEF (%)	58.2 ±1.8	56.3 ±1.7	14.9 ±1.1 ***	15.1 ±1.3 ***	9.5 ±0.9 ***	10.2 ±1.9 ***
CO (mL/min)	24.6 ±1.9	24.1 ±2.3	15.6 ±1.6 *	14.7 ±1.0 **	14.8 ±0.9 **	15.1 ±1.9 **
h (mm)	0.80 ±0.03	0.82 ±0.02	0.76 ±0.05	0.73 ±0.02	0.75 ±0.01	0.70 ±0.01
r (mm)	2.01 ±0.06	1.95 ±0.03	2.62 ±0.03 ***	2.49 ±0.01 ***	2.52 ±0.03 ***	2.54 ±0.05 ***
h/r	0.39 ±0.01	0.41 ±0.01	0.29 ±0.01 ***	0.29 ±0.01 ***	0.29 ±0.01 ***	0.27 ±0.02 ***
LV wall mass (mg)	83.3 ±2.2	81.7 ±4.7	113 ±12	96.5 ±4.6	98.3 ±4.9	104 ±14

Supplementary Table 3. Key resources

Reagent type (species) or resource	Designation	Source or reference	Identifiers	Additional information
antibody	anti-TRPM4 (rabbit polyclonal)	Novus Biologicals	Cat# NBP2-13487	Immunofluorescence (1:500)
antibody	anti-TRPM4 (rabbit polyclonal)	Alomone Labs	ACC-044	Western blot (1:200)
antibody	anti-PIEZO1 (mouse monoclonal)	Novus Biological	Cat# NBP2-76517	Immunofluorescence (1:200)
antibody	anti-PIEZO1 (rabbit polyclonal)	Alomone Labs	APC-087	Immunoprecipitation (0.85 ug)<br><br>Western blot (1:1000)
antibody	anti-integrin beta1 (rat monoclonal)	BD Biosciences	Cat# 550531	Immunofluorescence (1:200)
antibody	anti-CD31 (rat monoclonal)	BD Biosciences	Cat# 550274	Immunofluorescence (1:200)
antibody	anti-RFP (rabbit polyclonal)	Rockland	Cat# 600-401-379	Immunofluorescence (1:200)
antibody	goat anti-rat – AlexaFluor488	Invitrogen	Cat# A11006	Immunofluorescence (1:500)
antibody	goat anti-mouse – AlexaFluor647	Abcam	Cat# ab150119	Immunofluorescence (1:500)
antibody	donkey anti-rabbit – CF640	Biotium	Cat# 20178	Immunofluorescence (1:500)
antibody	donkey anti-mouse – AlexaFluor555	Invitrogen	Cat# A31570	Immunofluorescence (1:500)
Dye	wheat germ agglutinin – CF488A	Biotium	Cat # 29022	Immunofluorescence<br><br>(2 µg/ml)
Dye	phalloidin-FITC	Sigma	Cat# P5282	Immunofluorescence (5 µg/ml)
Dye	DAPI	Sigma	Cat# D9542	Immunofluorescence (0.1 µg/ml)

antibody	anti-mCherry (rat monoclonal)	Thermo Fisher Scientific	Cat# M11217	Western blot (1:500)
antibody	anti-CACNA1H (rabbit polyclonal)	Abcam	Cat# ab135974	Western blot (1:2000)
antibody	anti-NCX1 (rabbit polyclonal)	Thermo Fisher Scientific	Cat# PA5-104159	Western blot (1:1000)
antibody	anti-CaMKII delta (rabbit monoclonal)	Abcam	Cat# ab181052	Western blot (1:1000)
antibody	anti-p-CaMKII (Thr287) (rabbit polyclonal)	Thermo Fisher Scientific	Cat# PA5-37833	Western blot (1:5000)
antibody	anti-HDAC4 (rabbit monoclonal)	Cell Signaling Technology	Cat# 7628	Western blot (1:1500)
antibody	anti-p-HDAC4 (Ser632) (rabbit polyclonal)	Abcam	Cat# ab39408	Western blot (1:1500)
antibody	anti-MEF2A (rabbit polyclonal)	Cell Signaling Technology	Cat# 9736	Western blot (1:3000)
antibody	anti-NFATc4 (rabbit polyclonal)	Abcam	Cat# ab99431	Western blot (1:1500)
antibody	anti-GATA4 (mouse monoclonal)	Santa Cruz Biotechnology	Cat# sc-25310	Western blot (1:1000)
antibody	anti-SERCA2a (rabbit monoclonal)	Abcam	Cat# ab150435	Western blot (1:35000)
antibody	anti-phospholamban (rabbit monoclonal)	Abcam	Cat# ab219626	Western blot (1:1500)
antibody	Anti-phospho-phospholamban (Thr17) (rabbit polyclonal)	Badrilla	Cat# A010-13AP	Western blot (1:1500)
antibody	anti-CACNA1C	Abcam	Cat# ab84814	Western blot (1:10000)

	(mouse monoclonal			
antibody	anti-GAPDH (rabbit monoclonal)	Cell Signaling Technology	Cat# 2118	Western blot (1:10000)
antibody	anti-Histone H2B (rabbit polyclonal)	Abcam	Cat# ab1790	Western blot (1:5000)
antibody	goat anti-rabbit IgG (goat polyclonal)	Abcam	Cat# ab6721	Western blot (1:5000)
antibody	rabbit anti-mouse IgG (rabbit polyclonal)	Abcam	Cat# ab6728	Western blot (1:5000)
antibody	goat anti-rat IgG (goat polyclonal)	Abcam	Cat# ab97057	Western blot (1:5000)
sequence-based reagent	ANP (*Nppa*)_F	Sigma-Aldrich	PCR primers	TGATAGATGAAGGCAGGAAGCCGC
sequence-based reagent	ANP (*Nppa*)_R	Sigma-Aldrich	PCR primers	AGGATTGGAGCCCAGAGTGGACTAGG
sequence-based reagent	BNP (*Nppb*)_F	Sigma-Aldrich	PCR primers	TCTCCAGAGCAATTCAAGAT
sequence-based reagent	BNP (*Nppb*)_R	Sigma-Aldrich	PCR primers	AACAACTTCAGTGCGTTACA
sequence-based reagent	α-SA (*Acta1*)_F	Sigma-Aldrich	PCR primers	GTGAGATTGTGCGCGACATC
sequence-based reagent	α-SA (*Acta1*)_R	Sigma-Aldrich	PCR primers	GGCAACGGAAACGCTCATT
sequence-based reagent	Collagen III (*Col3A1*)_F	Sigma-Aldrich	PCR primers	GACAGATTCTGGTGCAGAGA
sequence-based reagent	Collagen III (*Col3A1*)_R	Sigma-Aldrich	PCR primers	CATCAACGACATCTTCAGGAAT

sequence-based reagent	*Piezo1* m112_R	Sigma-Aldrich	PCR primers	CTATGAGCCA CTGTTCACCAT
sequence-based reagent	*Piezo1* m112_F	Sigma-Aldrich	PCR primers	CTGCATGGCT AGTGGATAGG
sequence-based reagent	*Trpm4*_F	Sigma-Aldrich	PCR primers	GAGAAGCCCA CAGATGCCTA TG
sequence-based reagent	*Trpm4*_R	Sigma-Aldrich	PCR primers	AGCACCGACA CCACCAAGTT TG
sequence-based reagent	NCX1*(Slc8a1)*_F	Sigma-Aldrich	PCR primers	AAGAAACCGAA TGGAGAGAC
sequence-based reagent	NCX1*(Slc8a1)*_R	Sigma-Aldrich	PCR primers	TGGTCCCTCTC ATCAACTTC
sequence-based reagent	Cav3.2*(Cacna1h)* _F	Sigma-Aldrich	PCR primers	GTCCAGGATGC TCTCGCTAC
sequence-based reagent	Cav3.2*(Cacna1h)* _R	Sigma-Aldrich	PCR primers	GCGAGTGAGCA GAGGTGAC
sequence-based reagent	*Piezo1* KO_F	Sigma-Aldrich	PCR primers	CCAACAATACC AACTTGCAG
sequence-based reagent	*Piezo1* KO_R	Sigma-Aldrich	PCR primers	TGCAGGTGGTT CTGGATATAG
sequence-based reagent	*Piezo1* _F	Sigma-Aldrich	PCR primers	CTTGACCTGTC CCCTTCCCCAT CAAG
sequence-based reagent	*Piezo1* WT/fl_R	Sigma-Aldrich	PCR primers	CAGTCACTGCT CTTAACCATTGA GCCATCTC
sequence-based reagent	*Piezo1* P1KO_R	Sigma-Aldrich	PCR primers	AGGTTGCAGGG TGGCATGGCTC TTTTT
sequence-based reagent	Cav1.2(*Cacna1c*)_ F	Integrated DNA Technologies	PCR primers	TGACTACCTGA CTAGGGATTGG TCTA
sequence-based reagent	Cav1.2(*Cacna1c)* _R	Integrated DNA Technologies	PCR primers	TGCTCTAGGTT CCCTTCTGTTTT G
sequence-based reagent	*SERCA2a_F*	Integrated DNA Technologies	PCR primers	TAGCCAATGCA ATCGTGGGT

sequence-based reagent	*SERCA2a_R*	Integrated DNA Technologies	PCR primers	ACACTTTGCCC ATTTCAGGC
sequence-based reagent	*PLN_F*	Integrated DNA Technologies	PCR primers	TCAGGAGAGCC TCCACTATTGA
sequence-based reagent	*PLN_R*	Integrated DNA Technologies	PCR primers	TTAAGCTGAGT TGGCATGTTGC

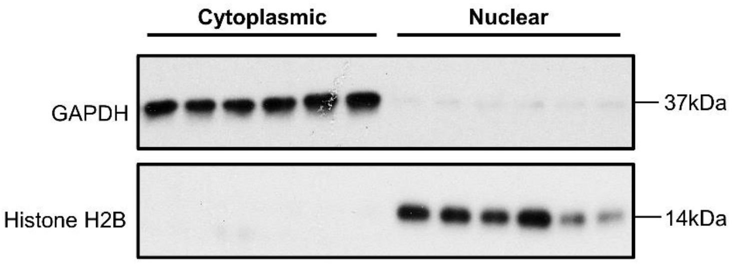

Supplementary Fig. 1. Demonstration of successful fractionation of subcellular compartments. The purity of the fractions extracted from the LV tissue was assessed by western blot using specific marker proteins: GAPDH for cytoplasmic fraction and Histone H2B for nuclear fraction. Each fraction (n = 6/group) was run side-by-side on the same blot and then probed separately against each of two primary antibodies: GAPDH and Histone H2B to validate fraction purity.

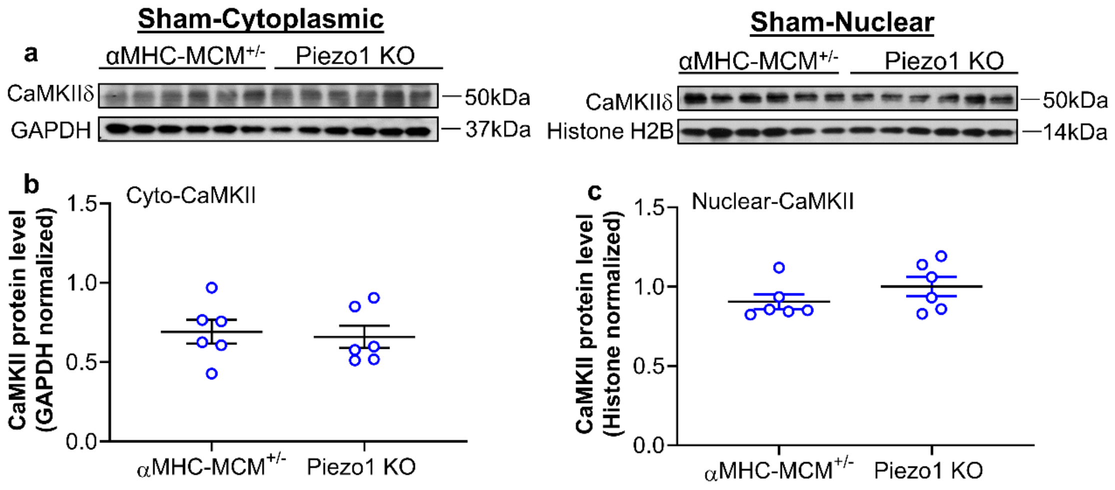

Supplementary Fig. 2. Demonstration of CaMKII protein levels of the fractionated cytoplasmic and nuclear compartments from sham operated αMHC-MCM$^{+/-}$ and Piezo1 KO hearts. (**a**) Representative western blots of CaMKII from cytoplasmic and nuclear fractions extracted from sham-operated α-MHC-MCM$^{+/-}$ and Piezo1 KO hearts, running side-by-side on the same blot and then probed separately against each of two primary antibodies: GAPDH and Histone H2B. (**b**,**c**) Quantitative densitometry of CaMKII protein expression was normalised to GAPGH (for cytoplasmic fraction) or Histone H2B (for nuclear fraction) from both sham-operated α-MHC-MCM$^{+/-}$ or sham-operated Piezo1 KO hearts (n = 6/group). Results are presented as mean ± SEM, Welch's T-test, two-tailed was used for **b**,**c**.

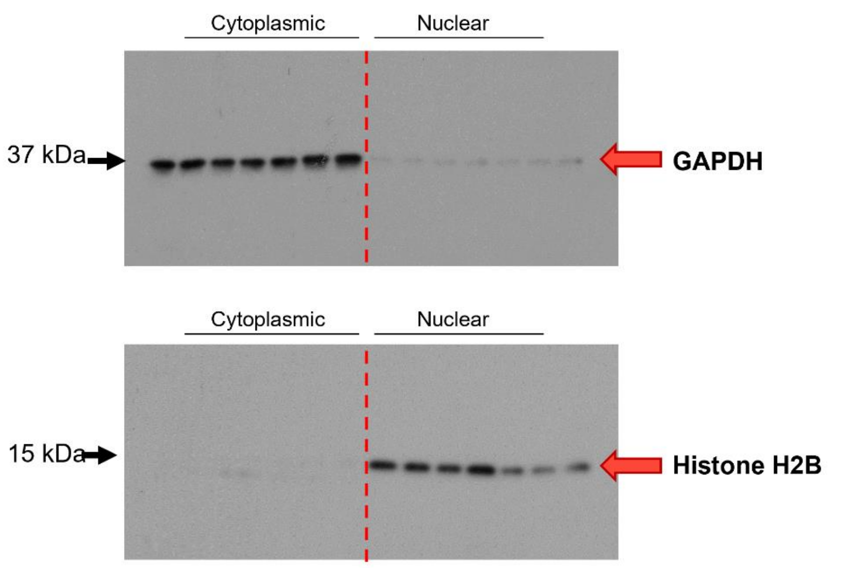

Additional Supplementary Fig. 1. Uncropped images of western blots, relating to Supplementary Fig. 1.

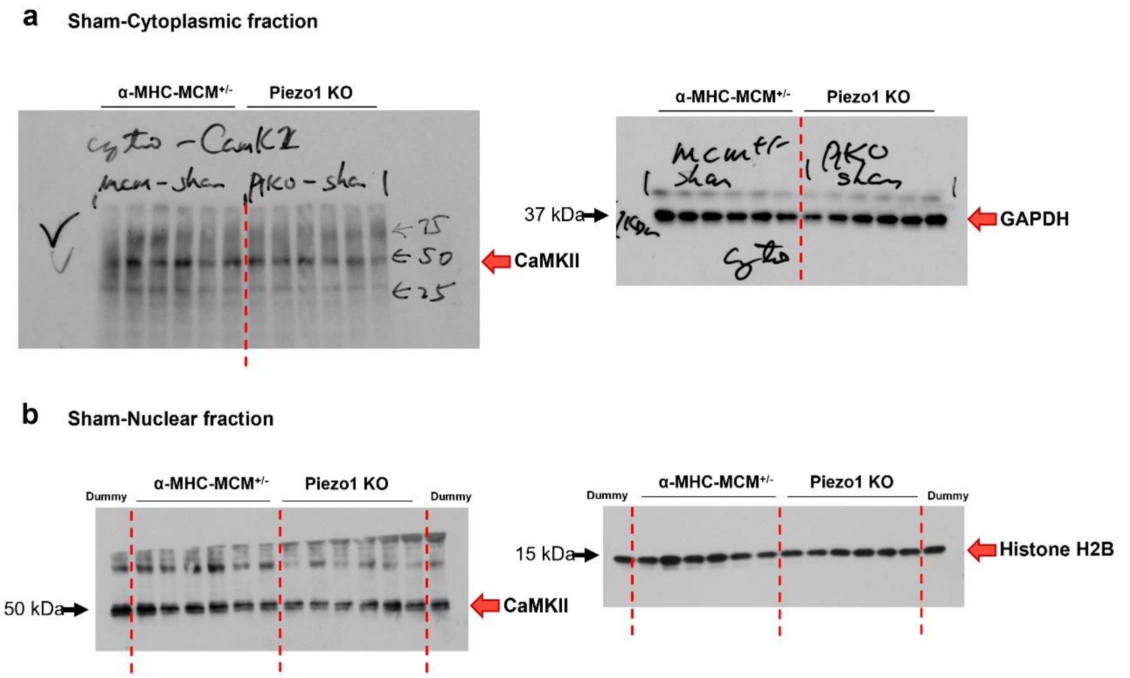

Additional Supplementary Fig. 2. Uncropped images of western blots, relating to Supplementary Fig. 2a.
