# Supplementary material for: Piezo1 is the cardiac mechanosensor that initiates the cardiomyocyte hypertrophic response to pressure overload in adult mice
Source: Nat Cardiovasc Res. 2022 Jun 13;1(6):577–91. doi: 10.1038/s44161-022-00082-0 (PMC11358016; doi:10.1038/s44161-022-00082-0)
Supplement: Supplementary file 2 — Reporting Summary [file 44161_2022_82_MOESM2_ESM.pdf]

nature portfolio

Corresponding author(s): Dr. Boris Martinac
Dr. Michael P. Feneley

Last updated by author(s): May 5, 2022

Reporting Summary

Nature Portfolio wishes to improve the reproducibility of the work that we publish. This form provides structure for consistency and transparency in reporting. For further information on Nature Portfolio policies, see our Editorial Policies and the Editorial Policy Checklist.

Statistics

For all statistical analyses, confirm that the following items are present in the figure legend, table legend, main text, or Methods section.

n/a	Confirmed	
☐	☒	The exact sample size (*n*) for each experimental group/condition, given as a discrete number and unit of measurement
☐	☒	A statement on whether measurements were taken from distinct samples or whether the same sample was measured repeatedly
☐	☒	The statistical test(s) used AND whether they are one- or two-sided<br>*Only common tests should be described solely by name; describe more complex techniques in the Methods section.*
☒	☐	A description of all covariates tested
☐	☒	A description of any assumptions or corrections, such as tests of normality and adjustment for multiple comparisons
☐	☒	A full description of the statistical parameters including central tendency (e.g. means) or other basic estimates (e.g. regression coefficient) AND variation (e.g. standard deviation) or associated estimates of uncertainty (e.g. confidence intervals)
☐	☒	For null hypothesis testing, the test statistic (e.g. *F*, *t*, *r*) with confidence intervals, effect sizes, degrees of freedom and *P* value noted<br>*Give P values as exact values whenever suitable.*
☒	☐	For Bayesian analysis, information on the choice of priors and Markov chain Monte Carlo settings
☒	☐	For hierarchical and complex designs, identification of the appropriate level for tests and full reporting of outcomes
☒	☐	Estimates of effect sizes (e.g. Cohen's *d*, Pearson's *r*), indicating how they were calculated

Our web collection on statistics for biologists contains articles on many of the points above.

Software and code

Policy information about availability of computer code

Data collection	1. NIS-Elements Microscope Imaging software, version 5.11.03 (Nikon Instruments, USA) was used for Ca2+ imaging.<br>2. ZEN Blue software, version 3.4.91.00000 (Carl Zeiss Microscopy, Germany) was used for immunofluorescence imaging.
Data analysis	1. GraphPad Prism software, version 7.04 (GraphPad, USA) was used for statistical analyses.<br>2. ImageJ software, version 1.52p (NIH; http://rsbweb.nih.gov/ij/) was used for histological and western blot data analysis.<br>3. NIS-Elements Microscope Imaging software, version 5.11.03 (Nikon Instruments, USA) was used for Ca2+ imaging data analysis.<br>4. ZEN Blue software, version 3.4.91.00000 (ZEISS, Germany) was used for immunofluorescence analysis.<br>5. Imaris software, version 9.5.1 (Oxford Instruments, UK) was used for 3D reconstruction of confocal images.

For manuscripts utilizing custom algorithms or software that are central to the research but not yet described in published literature, software must be made available to editors and reviewers. We strongly encourage code deposition in a community repository (e.g. GitHub). See the Nature Portfolio guidelines for submitting code & software for further information.

Data

Policy information about availability of data

All manuscripts must include a data availability statement. This statement should provide the following information, where applicable:

- Accession codes, unique identifiers, or web links for publicly available datasets
- A description of any restrictions on data availability
- For clinical datasets or third party data, please ensure that the statement adheres to our policy

All data generated or analysed during this study are included in this published article (and its supplementary information files).

Field-specific reporting

Please select the one below that is the best fit for your research. If you are not sure, read the appropriate sections before making your selection.

☒ Life sciences ☐ Behavioural & social sciences ☐ Ecological, evolutionary & environmental sciences

For a reference copy of the document with all sections, see nature.com/documents/nr-reporting-summary-flat.pdf

Life sciences study design

All studies must disclose on these points even when the disclosure is negative.

Sample size	The sample sizes were based on our previous published studies (Yu et al., 2021, PMID: 33659256; Guo et al., 2021, PMID: 34190686) and other studies in this field, which provide sufficient sample numbers to statistically detect differences between multiple experimental groups.
Data exclusions	No data were excluded.
Replication	Experiments were conducted repeatedly on different days, using different animals in a randomized order. As described in the Materials and Methods section, each sample/animal/well containing cells is considered a biological replicate. All experiments were conducted using at least two independent materials to reproduce similar results. We were able to produce similar results in the independent trials.<br>For each experiment, the exact n numbers are presented in the figure legends of the manuscript. The experimental data was analyzed statistically. For RT-PCR experiments, all biological replicates were run in technical triplicate as shown in the Materials and Methods section. All attempts at technical replication were successful for RT-PCR experiments. We also used existing protocols from our previous studies or other published studies and were able to reproduce similar results in the current study. These experiments include western blot for signaling pathway, RT-PCR for gene expression, and echocardiographic measurements for cardiac functions.
Randomization	All animals/samples were entered into the study in a randomized order.
Blinding	The investigators were blinded to the genotype/treatment during data collection and analysis. Full description is shown in the Materials and Methods section.

Reporting for specific materials, systems and methods

We require information from authors about some types of materials, experimental systems and methods used in many studies. Here, indicate whether each material, system or method listed is relevant to your study. If you are not sure if a list item applies to your research, read the appropriate section before selecting a response.

Materials & experimental systems

n/a	Involved in the study	
☐	☒	Antibodies
☐	☒	Eukaryotic cell lines
☒	☐	Palaeontology and archaeology
☐	☒	Animals and other organisms
☒	☐	Human research participants
☒	☐	Clinical data
☒	☐	Dual use research of concern

Methods

n/a	Involved in the study	
☒	☐	ChIP-seq
☒	☐	Flow cytometry
☒	☐	MRI-based neuroimaging

Antibodies

Antibodies used

1. anti-TRPM4 (rabbit polyclonal), Novus Biologicals, Cat# NBP2-13487
2. anti-TRPM4 (rabbit polyclonal), Alomone Labs, Cat# ACC-044
3. anti-PIEZO1 (mouse monoclonal), Novus Biologicals, Cat# NBP2-75617, Clone: 2-10
4. anti-PIEZO1 (rabbit polyclonal), Alomone Labs, APC-087
5. anti-mouse CD29 (beta1 integrin) (rat monoclonal), BD Biosciences, Cat# 550531, Clone: 9EG7
6. anti-CD31 (rat monoclonal), BD Biosciences, Cat# 550274, Clone: MEC 13.3
7. anti-RFP (rabbit polyclonal), Rockland, Cat# 600-401-379
8. anti-mCherry (rat monoclonal), Thermo Fisher Scientific, Cat# M11217, Clone: 16D7
9. anti-CACNA1H (rabbit polyclonal), Abcam, Cat# ab135974
10. anti-NCX1 (rabbit polyclonal), Thermo Fisher Scientific, Cat# PA5-104159
11. anti-CaMKII delta (rabbit monoclonal), Abcam, Cat# ab181052, Clone: EPR13095
12. anti-p-CaMKII (Thr287)(rabbit polyclonal), Thermo Fisher Scientific, Cat# PA5-37833
13. anti-HDAC4 (rabbit monoclonal), Cell Signaling Technology, Cat# 7628, Clone: D15C3
14. anti-p-HDAC4 (Ser632) (rabbit polyclonal), Abcam, Cat# ab39408
15. anti-MEF2A (rabbit polyclonal), Cell Signaling Technology, Cat# 9736

	16. anti-NFATc4 (rabbit polyclonal), Abcam, Cat# ab99431<br>17. anti-GATA4 (mouse monoclonal), Santa Cruz Biotechnology, Cat# sc-25310, Clone: G-4<br>18. anti-SERCA2a (rabbit monoclonal), Abcam, Cat# ab150435, Clone: EPR9392<br>19. anti-phospholamban (rabbit monoclonal), Abcam, Cat# ab219626, Clone: EPR21897<br>20. anti-phospho-phospholamban (Thr17) (rabbit polyclonal), Badrilla, Cat# A010-13AP<br>21. anti-CACNA1C (mouse monoclonal), Abcam, Cat# ab84814, Clone: L57/46<br>22. anti-GAPDH (rabbit monoclonal), Cell Signaling Technology, Cat# 2118, Clone: 14C10<br>23. anti-Histone H2B (rabbit polyclonal), Abcam, Cat# ab1790<br>24. goat anti-rabbit IgG (goat polyclonal), Abcam, Cat# ab6721<br>25. rabbit anti-mouse IgG (rabbit polyclonal), Abcam, Cat# ab6728<br>26. goat anti-rat IgG (goat polyclonal), Abcam, Cat# ab97057<br>27. goat anti-rat – AlexaFluor488, Invitrogen Cat# A11006<br>28. goat anti-mouse – AlexaFluor647, Abcam, Cat# ab150119<br>29. donkey anti-rabbit – CF640, Biotium, Cat# 20178<br>30. donkey anti-mouse – AlexaFluor555, Invitrogen, Cat# A31570
Validation	1. anti-TRPM4 (rabbit polyclonal), Novus Biologicals, Cat# NBP2-13487, immunofluorescence (1:500), orthogonal validation in human tissue.<br>2. anti-TRPM4 (rabbit polyclonal), Alomone Labs, ACC-044, western blot (1:200), KO validated in human, rat, and mouse samples, PMID: 34190686.<br>3. anti-PIEZO1 (mouse monoclonal), Novus Biological, Cat# NBP2-75617, immunofluorescence (1:200), use in EM reported in scientific literature (PMID:34489534). PIEZO1 antibody validated for WB from a verified customer review.<br>4. anti-PIEZO1 (rabbit polyclonal), Alomone Labs, APC-087, immunoprecipitation (0.85 ug)/western blot (1:1000), KO validated in mouse samples for immunostaining (PMID: 29712913). WB citation: PMID: 29735991.<br>5. anti-integrin beta1 (rat monoclonal), BD Biosciences, Cat# 550531, immunofluorescence (1:200), PMID: 7692444.<br>6. anti-CD31 (rat monoclonal), BD Biosciences, Cat# 550274, immunofluorescence (1:200), PMID: 35087060.<br>7. anti-RFP (rabbit polyclonal), Rockland, Cat# 600-401-379, immunofluorescence (1:200), PMID: 35172139.<br>8. anti-mCherry (rat monoclonal), Thermo Fisher Scientific, Cat# M11217, western blot (1:500), PMID: 34853300.<br>9. anti-CACNA1H (rabbit polyclonal), Abcam, Cat# ab135974, western blot (1:2000), PMID: 34917013.<br>10. anti-NCX1 (rabbit polyclonal), Thermo Fisher Scientific, Cat# PA5-104159, western blot (1:1000). No official or published validation.<br>11. anti-CaMKII delta (rabbit monoclonal), Abcam, Cat# ab181052, western blot (1:1000), KO validated in HEK-293T cell line. WB citation: PMID: 34190686.<br>12. anti-p-CaMKII (Thr287)(rabbit polyclonal), Thermo Fisher Scientific, Cat# PA5-37833, western blot (1:5000), validated by cell treatment in SK-N-AS cell line. WB citation: PMID: 34190686.<br>13. anti-HDAC4 (rabbit monoclonal), Cell Signaling Technology, Cat# 7628, western blot (1:1500), PMID: 35169129.<br>14. anti-p-HDAC4 (Ser632) (rabbit polyclonal), Abcam, Cat# ab39408, western blot (1:1500), PMID: 27131508.<br>15. anti-MEF2A (rabbit polyclonal), Cell Signaling Technology, Cat# 9736, western blot (1:3000), PMID: 34190686.<br>16. anti-NFATc4 (rabbit polyclonal), Abcam, Cat# ab99431, western blot (1:1500), PMID: 34190686.<br>17. anti-GATA4 (mouse monoclonal), Santa Cruz Biotechnology, Cat# sc-25310, western blot (1:1000), PMID: 34190686.<br>18. anti-SERCA2a (rabbit monoclonal), Abcam, Cat# ab150435, western blot (1:35000), PMID: 34462437.<br>19. anti-phospholamban (rabbit monoclonal), Abcam, Cat# ab219626, western blot (1:1500), PMID: 35111375.<br>20. anti-phospho-phospholamban (Thr17) (rabbit polyclonal), Badrilla, Cat# A010-13AP, western blot (1:1500), PMID: 33593074.<br>21. anti-CACNA1C (mouse monoclonal), Abcam, Cat# ab84814, western blot (1:10000), PMID: 35118141.<br>22. anti-GAPDH (rabbit monoclonal), Cell Signaling Technology, Cat# 2118, western blot (1:10000), orthogonal validation. WB citation PMID: 34190686.<br>23. anti-Histone H2B (rabbit polyclonal), Abcam, Cat# ab1790, western blot (1:5000), PMID: 34588981.

Eukaryotic cell lines

Policy information about cell lines

Cell line source(s)	The H9c2 cells were a kind gift from Prof Richard Harvey at the Victor Chang Cardiac Research Institute, Australia. Original commercial source: American Type Culture Collection, USA.
Authentication	The cell line was not authenticated specifically in this study.
Mycoplasma contamination	The cell line was tested negative for mycoplasma contamination.
Commonly misidentified lines<br>(See ICLAC register)	No commonly misidentified cell line was used in the study.

Animals and other organisms

Policy information about studies involving animals; ARRIVE guidelines recommended for reporting animal research

Laboratory animals	All the mice used for experiments in this study are C57BL/6J strain, male, 8-13 weeks old.<br>The homozygous Piezo1 reporter mice expressing a fusion protein of Piezo1 and the fluorophore TdTomato (Piezo1P1-tdT/P1-tdT, The Jackson Laboratory Stock, No: 029214) were backcrossed to C57BL/6J mice to yield heterozygous Piezo1P1-tdT/wt mice that were intercrossed to each other to obtain homozygous Piezo1P1-tdT/P1-tdT mice for the experiments, and their WTLs served as controls.

	To generate inducible cardiomyocyte-specific Piezo1 knockout (KO) mice, we crossed homozygous Piezo1flox/flox mice (The Jackson Laboratory Stock, No: 029213) with homozygous Myh6-MerCreMer mice (MCM), which have a tamoxifen-inducible Cre recombinase under the control of the α-myosin heavy-chain (αMHC; Myh6) promoter20, to produce Piezo1flox/flox; αMHC-MCM+/- (termed P1fl/flMCM+/-) mice. The age and sex-matched Piezo1wt/wt;αMHC-MCM+/- (termed αMHC-MCM+/-) mice and Piezo1wt/wt; αMHC-MCM-/- (termed P1wt/wtMCM-/-) mice were used as controls for experiments characterizing phenotype at baseline.
Wild animals	This study did not involve wild animals.
Field-collected samples	This study did not involve samples collected from the field.
Ethics oversight	All experimental procedures were approved by the Animal Ethics Committee of Garvan/St Vincent's (Australia), in accordance with the guidelines of both the Australian code for the care and use of animals for scientific purposes (8th edition, National Health and Medical Research Council, AU, 2013) and the Guide for the Care and Use of Laboratory Animals (8th edition, National Research Council, USA, 2011).

Note that full information on the approval of the study protocol must also be provided in the manuscript.
