# Supplementary figures and images for: Piezo1 is the cardiac mechanosensor that initiates the cardiomyocyte hypertrophic response to pressure overload in adult mice
Source: Nat Cardiovasc Res. 2022 Jun 13;1(6):577–91. doi: 10.1038/s44161-022-00082-0 (PMC11358016; doi:10.1038/s44161-022-00082-0)

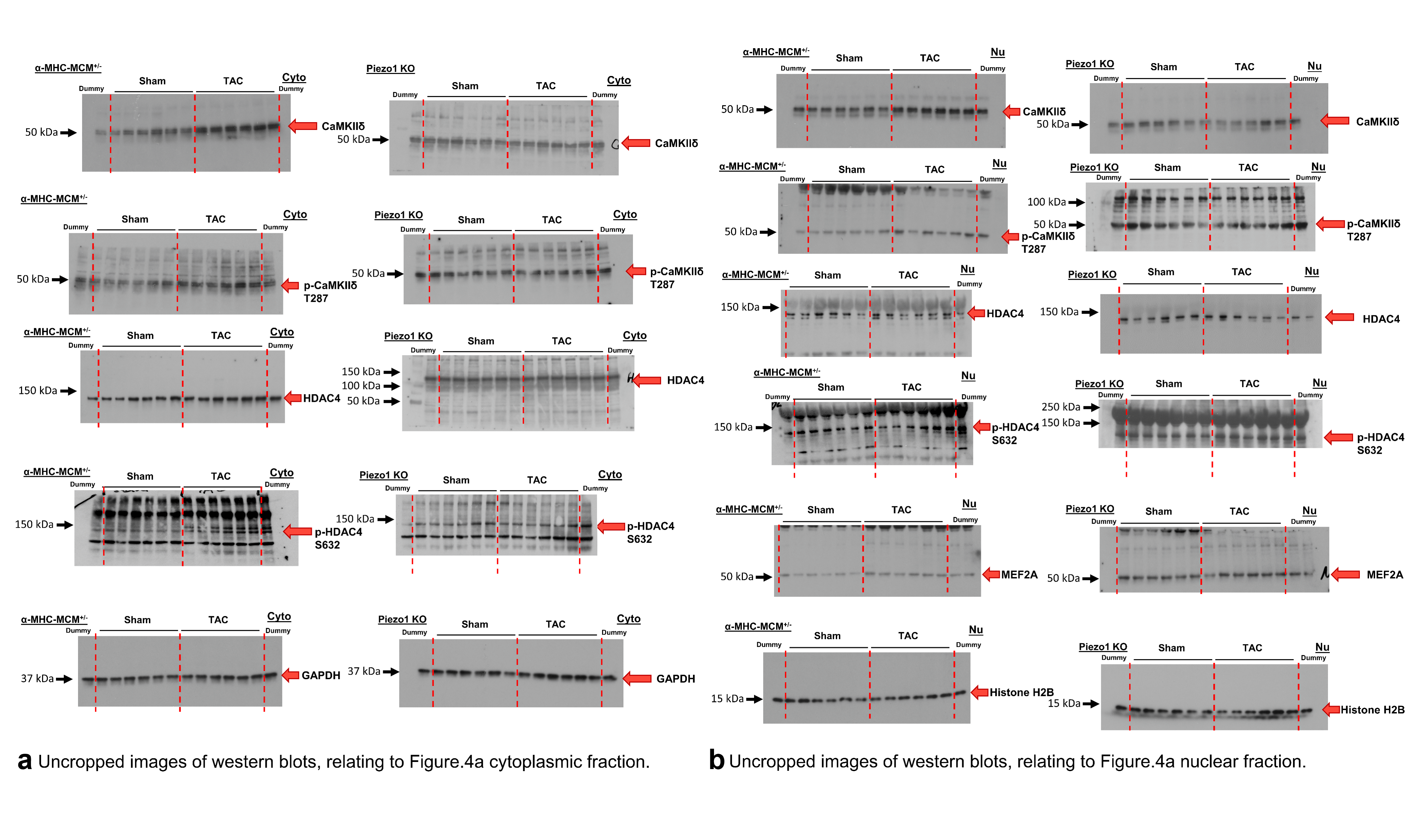

Supplement: Supplementary file 9 — Full-length, unprocessed blots. [file 44161_2022_82_MOESM9_ESM.tif]
